# Mitochondrial Quality Control Links Exercise to Sterile Inflammation in the Cardiovascular System: A Narrative Review

**DOI:** 10.3390/antiox15091134

**Published:** 2026-09-07

**Authors:** Ying Wen, Pengfei Zhang, Xinyu Liao, Jiankang Liu, Yang Zhang, Xuyun Liu

**Affiliations:** 1 School of Medicine, Northwest University, Xi’an 710069, China; wenying2104@stumail.nwu.edu.cn; 2 Key Laboratory of Ministry of Education, School of Aerospace Medicine, Fourth Military Medical University, Xi’an 710032, China; zhangpengfei@fmmu.edu.cn; 3 Center for Mitochondrial Biology and Medicine, The Key Laboratory of Biomedical Information Engineering of Ministry of Education, School of Life Science and Technology, Xi’an Jiaotong University, Xi’an 710049, China; liaoxinyu2026@stu.xjtu.edu.cn; 4 Qingdao Municipal Hospital, Shandong Engineering Research Center of Precision Intervention for Aging, Shandong Key Laboratory of Neurorehabilitation, School of Life Sciences and Health, University of Health and Rehabilitation Sciences, Qingdao 266113, China; jkliu@uhrs.edu.cn; 5 Department of Dermatology, The First Affiliated Hospital of Xi’an Jiaotong University, Xi’an 710061, China; 6 Department of Medical Electronics, School of Biomedical Engineering, Air Force Medical University, Xi’an 710032, China

**Keywords:** exercise, inflammation, mitochondrial DAMPs, mitochondrial quality control, cardiovascular system

## Abstract

Preservation of mitochondrial integrity has emerged as a central hub in the anti-inflammatory effect of exercise. This narrative review advances a framework in which mitochondrial damage-associated molecular patterns (mtDAMPs) serve as the mechanistic bridge between exercise and inflammation. Mitochondrial dysfunction releases mtDAMPs, including mitochondrial DNA (mtDNA), reactive oxygen species, cardiolipin, N-formyl peptides, and ATP, which activate cGAS–STING, the NLRP3 inflammasome, TLR9, AIM2, ZBP1, and NF-κB signaling. Crosstalk among these pathways allows mild mitochondrial damage to escalate into chronic inflammation. Exercise opposes this cascade through the AMPK–PGC-1α axis, which coordinately activates four mitochondrial quality control (MQC) modules: biogenesis, antioxidant defense, dynamics, and mitophagy. The cardiovascular system illustrates this framework, as myocardial inflammation runs mainly through mtDNA–cGAS–STING signaling and vascular inflammation through oxidized mtDNA–NLRP3 signaling, while cardiovascular aging engages both axes at once. Throughout, exercise refers to repeated training rather than to a single bout, and the framework targets middle-aged and older adults with, or at risk of, cardiovascular disease. The upstream half of the sequence, in which training raises mitochondrial content and antioxidant capacity, rests on human muscle biopsy data; the downstream half remains largely preclinical. MQC is therefore proposed as a testable target rather than an established one.

## 1. Introduction

Inflammation is a fundamental component of the innate immune response that serves to defend against pathogens and facilitate tissue repair [1]. However, the persistence of the process can evolve into a state of low-grade inflammation, which is characterized by a sustained, subclinical elevation of circulating pro-inflammatory factors. This condition has been increasingly recognized as a common pathogenic driver underlying a wide spectrum of non-communicable diseases, including cardiovascular disease, type 2 diabetes, cancer and neurodegenerative disorders, and is independently associated with elevated all-cause mortality [2]. For instance, it is reported that individuals with higher aggregated inflammation scores exhibited a significantly increased risk of cardiometabolic multimorbidity and an accelerated onset of disease compared with those in the lowest inflammation category [3]. Cardiovascular disease carries the largest share of this burden and is the setting in which the mechanisms discussed below have been characterized in most detail, and we therefore use it as the focus of this review.

Mitochondria have long been regarded as the primary sites of energy production in eukaryotic cells. Emerging evidence over the past decade has revealed their critical role as central regulators of innate immune signaling [4,5]. Owing to their endosymbiotic origin from an ancestral α-proteobacterium, mitochondria retain several bacteria-like molecular features, including a circular genome enriched in unmethylated CpG motifs (mitochondrial DNA, mtDNA), N-formylated peptides, and the distinctive phospholipid cardiolipin [6,7]. When mitochondrial dysfunction occurs, whether through oxidative damage, membrane permeabilization, or impaired quality control, these components can be released into the cytosol or the extracellular space, where they are recognized by pattern recognition receptors as mitochondrial damage-associated molecular patterns (mtDAMPs), thereby triggering potent inflammatory responses [5,7]. Among the major mtDAMPs identified to date, mtDNA activates both the cyclic GMP-AMP synthase–stimulator of interferon genes (cGAS-STING) pathway and the NOD-like receptor family pyrin domain containing 3 (NLRP3) inflammasome pathway; mitochondrial reactive oxygen species (mtROS) serve as direct activators of the NLRP3 inflammasome and nuclear factor kappa-light-chain-enhancer of activated B cells (NF-κB) signaling [5,8,9]. These findings collectively indicate that mitochondrial dysfunction engages a multi-pathway inflammatory network mediated by diverse mtDAMPs, producing a cumulative and amplified inflammatory response [5]. Both the heart and the vessel wall are unusually exposed to this problem. Cardiomyocytes carry one of the highest mitochondrial volume densities of any cell type, and endothelial and smooth muscle cells sit at the interface between mechanical load and redox signaling, so the threshold at which mitochondrial damage becomes an immune signal is reached more readily in these tissues than in most others.

Exercise has been described as a polypill because of the breadth of its health benefits [10]. Its relationship with inflammation is less simple than that description suggests, and the distinction matters for the argument developed here. A single bout of exercise raises circulating interleukin-6 (IL-6), and this muscle-derived IL-6 in turn induces IL-1 receptor antagonist and IL-10 [11,12]. IL-6 released from contracting muscles therefore behaves as a myokine rather than as a marker of disease, and its time course after a bout is the reverse of what is seen in chronic inflammation. Training has the opposite effect on the resting state. A meta-analysis of 83 controlled trials in 3769 participants found that training lowers resting C-reactive protein (CRP) levels, an effect that is larger when weight loss occurs but persists without it [13]. Throughout this review, the anti-inflammatory effect of exercise refers to this training-induced lowering of the resting inflammatory set point, and where a finding comes from a single bout, it is indicated. Despite this well-established anti-inflammatory efficacy, the precise molecular mechanisms by which exercise suppresses the initiation and propagation of inflammatory signaling at the subcellular level remain incompletely understood. A substantial body of evidence has demonstrated that regular exercise activates the AMP-activated protein kinase (AMPK)/peroxisome proliferator-activated receptor gamma coactivator 1-alpha (PGC-1α) signaling axis, thereby comprehensively enhancing the mitochondrial quality control (MQC) system [14,15]. MQC is a coordinated network of cellular processes that maintains mitochondrial integrity and function, encompassing mitochondrial biogenesis (the synthesis of new mitochondria), mitochondrial dynamics (the balance between fusion and fission that regulates organelle morphology and content exchange), mitophagy (the selective autophagic removal of damaged mitochondria), and antioxidant defense mechanisms [16,17]. From a broader mechanistic perspective, if exercise enhances MQC to maintain mitochondrial health, and mitochondrial dysfunction drives multi-pathway inflammation through mtDAMPs release as discussed above, then the anti-inflammatory effect of exercise can be understood from a mitochondrial perspective: exercise augments MQC capacity, thereby systematically reducing the generation and release of various mtDAMPs, which in turn simultaneously suppresses multiple downstream innate immune inflammatory pathways, including cGAS-STING, NLRP3, Toll-like receptor 9 (TLR9), and NF-κB [5,18,19]. This review advances a unifying framework in which mtDAMPs serve as the mechanistic bridge between exercise and inflammation.

Our aim is narrower than a survey of how exercise lowers inflammation, which has been covered elsewhere. We ask whether a single upstream node, mitochondrial quality control, can account for the simultaneous restraint of innate immune pathways that are otherwise mechanistically distinct, and whether the cardiovascular system offers a fair test of that idea. Three boundaries define the scope. In terms of outcome, we address chronic sterile inflammation of the myocardium and vessel walls, rather than the systemic acute-phase response that follows a single bout or infection-driven inflammation. In terms of population, the framework is developed for middle-aged and older adults who have, or are at risk of, cardiovascular disease, since this is the group in which a raised resting inflammatory set point carries prognostic weight and in which exercise is prescribed as treatment; findings from young volunteers and from athletes are used only where they establish mechanism and are identified as such. In terms of intervention, exercise refers throughout to repeated training over weeks to months rather than to a single bout, and claims are attached to a modality and an intensity wherever the primary literature allows. If mtDAMP release rather than circulating cytokine concentration is the proximal readout of this mechanism, then exercise and mitochondria-targeted agents become comparable on a single scale, and the question of how best to combine them becomes an empirical one.

### Search Strategy and Selection Criteria

This article is a narrative review and is reported as one. No protocol was registered, and no PRISMA flow diagram is presented, because the question asked here is mechanistic rather than quantitative: the evidence runs from cell culture through rodent models to human trials, and those bodies of work are not commensurable under a single set of inclusion criteria. What follows is an account of how the literature was identified, so that readers can judge the coverage for themselves.

We searched PubMed from inception to August 2026. The search combined terms for the mitochondrial side (mitochondrial DAMP, mtDNA, cardiolipin, mitochondrial ROS, mitochondrial quality control, mitophagy, mitochondrial biogenesis, mitochondrial dynamics), the innate immune side (cGAS-STING, NLRP3, TLR9, AIM2, sterile inflammation) and the exercise side (exercise, physical activity, endurance training, resistance training), each crossed with cardiovascular, myocardium, endothelium, atherosclerosis and aging. Reference lists of the retrieved articles were screened by hand, and the searches were repeated before submission so that recent work was not missed.

An article was used when it reported original data bearing on one of the three links in the framework—that is, exercise to mitochondrial quality control, mitochondrial quality control to mtDAMP release, or mtDAMP to innate immune activation in cardiovascular tissue—when it was written in English, and when the full text was available. Reviews were retained for background statements and for consensus definitions, and every mechanistic claim in the text is referenced to the study that established it rather than to a review of that study. Where a claim rests on animal or cell work alone, the species or model is named in the sentence that makes it.

The Scale for the Assessment of Narrative Review Articles was used as an internal check while the manuscript was revised. Its authors state that it is an instrument for critical appraisal rather than a reporting guideline [20], and it is cited here in that spirit, as a self-assessment aid and not as a standard the article claims to meet.

## 2. Mitochondrial Dysfunction Triggers Inflammatory Response Through mtDAMPs

### 2.1. Mitochondrial Dysfunction and the Release of mtDAMPs

Multiple pathological factors, including the progressive accumulation of mtDNA mutations during aging, electron transport chain (ETC) overload caused by substrate excess in metabolic diseases, and the decline in mitochondrial quantity and quality resulting from sedentary lifestyles, can drive mitochondrial dysfunction [17,21]. The core manifestations of mitochondrial dysfunction include decreased ETC efficiency leading to insufficient ATP production, pathological opening of the mitochondrial permeability transition pore (mPTP), and subsequent collapse of mitochondrial membrane potential (ΔΨm), as well as mitochondrial outer membrane permeabilization (MOMP) mediated by BCL-2-associated X protein (BAX)/BCL-2 homologous antagonist killer (BAK) macropores and voltage-dependent anion channel (VDAC) oligomerization [21,22,23]. These events, acting individually or cooperatively, lead to the disruption of mitochondrial membrane integrity [23,24]. When mitochondrial membrane integrity is compromised, various molecular components that are normally compartmentalized within the mitochondrial double-membrane system, such as mtDNA, unique membrane lipids (e.g., cardiolipin), ROS, and N-formylated proteins [25], can be released into the cytosol through multiple pathways, including mPTP, BAX/BAK macropores, VDAC oligomeric pores, and gasdermin D (GSDMD) pores [23,24]. Once released, these components are recognized as “danger signals” by pattern recognition receptors (PRRs) of the host innate immune system [26,27]. These released components are collectively termed mtDAMPs. The major mtDAMPs identified to date include mtDNA, mtROS, cardiolipin, N-formyl peptides, and ATP [27,28,29,30]. Among these, mtDNA and mtROS have been the most extensively studied.

### 2.2. mtDNA-Activated Innate Immune Signaling Pathways

The immune consequences of cytosolic mtDNA depend on the form in which it reaches the cytosol; of these, BAX/BAK-mediated MOMP during apoptosis is the canonical and best-characterized, releasing intact nucleoids. The contents released from the BAX/BAK pore include intact mtDNA nucleoids that remain bound to TFAM and other nucleoid-associated proteins [31,32]. Under normal apoptosis, this signal stays silent because activated caspases cleave cGAS and interferon regulatory factor 3 (IRF3); it becomes immunogenic only when caspase activity is lost or inhibited [33,34]. In the cytosol, cGAS senses mtDNA, and its sensitivity scales with DNA length. Upon activation, cGAS undergoes conformational changes and catalyzes the synthesis of the second messenger 2′3′-cyclic guanosine monophosphate–adenosine monophosphate (2′3′-cGAMP) [35]. This dinucleotide subsequently binds to and activates the endoplasmic reticulum (ER)-resident adaptor protein STING [36]. Activated STING recruits and activates the kinase TANK-binding kinase 1 (TBK1), which phosphorylates STING itself; phosphorylated STING then binds and presents IRF3 for TBK1-mediated phosphorylation, driving expression of type I interferons (IFN-I) and interferon-stimulated genes (ISGs) [37]. STING also drives a parallel NF-κB branch. In myeloid cells, this branch is mediated redundantly by TBK1 and IKKε and is less sensitive to TBK1 inhibition than IRF3 activation, which is why the two arms can be uncoupled experimentally [38]. An endosomal route also feeds this pathway: under mtDNA replication stress, enlarged nucleoids are trafficked into endosomes for disposal, and rupture during transit releases mtDNA into the cytosol [39].

The NLRP3 inflammasome is a multiprotein complex comprising the sensor protein NLRP3, the adaptor apoptosis-associated speck-like protein containing a CARD (ASC), and the effector pro-caspase-1, whose activation follows a classical two-signal model. Signal 1, priming, acts through NF-κB-dependent upregulation of NLRP3 expression, which is the rate-limiting checkpoint for subsequent activation, together with transcription of pro-IL-1β; signal 2 triggers NLRP3 oligomerization and ASC speck formation [40]. Newly synthesized mtDNA, which lacks sufficient TFAM packaging and protection, is susceptible to oxidative damage by mtROS [9,41]. Within the mitochondrial matrix, oxidized mtDNA (ox-mtDNA) is either repaired by the DNA glycosylase 8-oxoguanine DNA glycosylase 1 (OGG1) or cleaved by the endonuclease flap endonuclease 1 (FEN1) into approximately 500–650 bp fragments [41]. These ox-mtDNA fragments are then released into the cytosol through the coordinated action of two structurally and functionally independent but synergistic channel systems: mPTP across the inner membrane and VDAC oligomers across the outer membrane [41,42]. Once in the cytosol, ox-mtDNA fragments bind NLRP3 as a specific activation signal (signal 2) and, in concert with the NF-κB-mediated priming signal (signal 1), activate the NLRP3 inflammasome, leading to caspase-1-dependent maturation and secretion of the pro-inflammatory cytokines IL-1β and IL-18 [8,41].

Furthermore, mtDNA can enter the endosomal compartment through multiple routes, including endocytosis of extracellular free mtDNA, uptake of mitochondria-derived vesicles (MDVs) or mtDNA-containing extracellular vesicles, and shuttling of mtDNA into endolysosomal compartments via defective mitophagy or autophagy [5,43,44]. Upon reaching the endosome, mtDNA engages TLR9, which recognizes hypomethylated CpG motifs characteristic of mtDNA, and initiates a myeloid differentiation factor 88 (MyD88)-dependent signaling cascade that activates NF-κB and IRF7, ultimately inducing pro-inflammatory gene expression and type I interferon production [5,45]. Additionally, the AIM2 (absent in melanoma 2) inflammasome detects cytosolic double-stranded DNA, including mtDNA, through its HIN200 domain and recruits ASC to activate caspase-1 [46,47]. Z-DNA binding protein 1 (ZBP1) senses Z-form mtDNA and, together with cGAS, receptor-interacting protein kinase 1, and RIPK3, sustains type I interferon signaling. This route was characterized in the doxorubicin-treated mouse heart and is therefore directly relevant to cardiotoxicity, although it has not been examined in the context of exercise [48].

**Figure 1:**
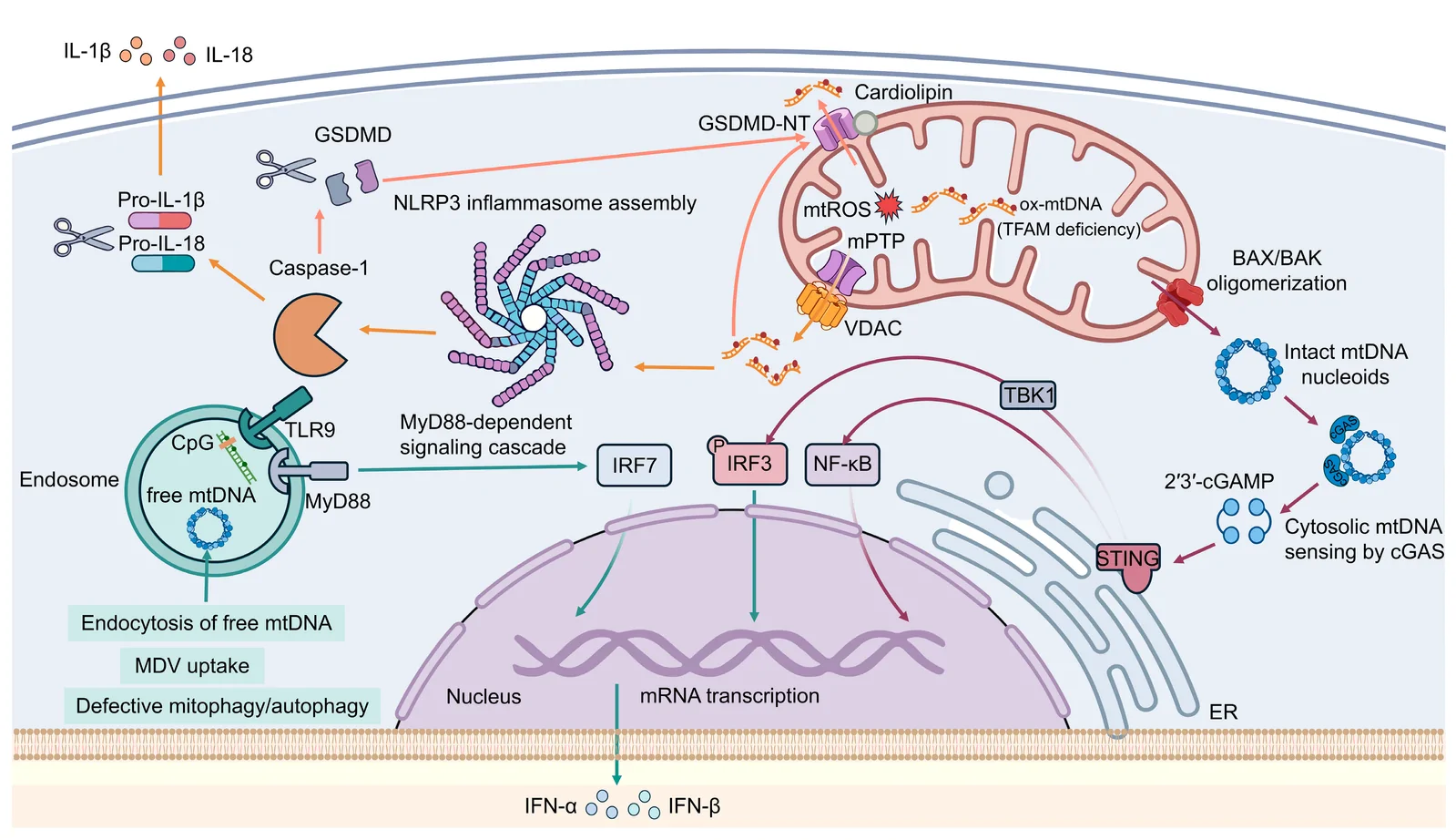
mtDNA-activated innate immune signaling pathways. Damaged mitochondria release mitochondrial DNA (mtDNA) into three compartments that engage distinct innate immune receptors. Free mtDNA enters the endosome via endocytosis, mitochondria-derived vesicle (MDV) uptake, or defective mitophagy/autophagy, where its CpG motifs activate TLR9 signaling. In the matrix, mtROS oxidize newly synthesized mtDNA under TFAM deficiency, and the resulting ox-mtDNA fragments exit through mPTP and VDAC oligomers to activate the NLRP3 inflammasome. Apoptotic BAX/BAK oligomerization releases intact, TFAM-bound mtDNA nucleoids that activate cGAS-STING signaling. Arrow colors mark the three routes: green, endosomal TLR9 signaling; orange, cytosolic ox-mtDNA sensing by NLRP3; dark red, cGAS-STING signaling from cytosolic mtDNA nucleoids; salmon, the GSDMD-NT feedback that permeabilizes mitochondria and releases further mtDNA. Abbreviations: BAX/BAK, BCL-2-associated X protein/BCL-2 homologous antagonist killer; cGAS, cyclic GMP-AMP synthase; 2′3′-cGAMP, 2′3′-cyclic GMP-AMP; ER, endoplasmic reticulum; IRF, interferon regulatory factor; MDV, mitochondria-derived vesicle; mPTP, mitochondrial permeability transition pore; mtROS, mitochondrial reactive oxygen species; MyD88, myeloid differentiation factor 88; NF-κB, nuclear factor kappa B; NLRP3, NOD-like receptor family pyrin domain containing 3; ox-mtDNA, oxidized mtDNA; STING, stimulator of interferon genes; TBK1, TANK-binding kinase 1; TFAM, mitochondrial transcription factor A; TLR9, Toll-like receptor 9; VDAC, voltage-dependent anion channel.

Collectively, these multiple innate immune pathways activated by mtDNA are fundamentally governed by the nature and severity of mitochondrial membrane damage, which determines the form of mtDNA released and thereby tends to enable graded immune regulation (Figure 1). Mild damage tends to engage the TLR9 pathway through MDVs or defective autophagy-mediated delivery of free mtDNA, initiating immune surveillance. Moderate damage preferentially activates the NLRP3 pathway via ox-mtDNA fragments, in conjunction with TLR9 signaling, to mediate localized inflammation. Severe damage, such as apoptotic MOMP, triggers the release of intact mtDNA nucleoids to activate the cGAS-STING pathway, superimposed upon NLRP3, TLR9, AIM2, and ZBP1 signaling. Through downstream effector complementarity and NF-κB signal cross-amplification, mtDNA as a single DAMP molecule achieves coordinated regulation of multiple innate immune pathways, enabling a precise and graded immune response to mitochondrial stress and membrane integrity loss [47]. However, it should be noted that this graded model remains a conceptual framework, as the activation of these pathways exhibits substantial cross-talk and tissue specificity across different pathological contexts.

Tissue specificity is central to what follows. In Section 4, we show that the myocardium and the vessel wall sit at different points of this graded scheme, and that this is why a single upstream intervention can restrain both.

### 2.3. mtROS-Mediated Inflammatory Signaling

**Figure 2:**
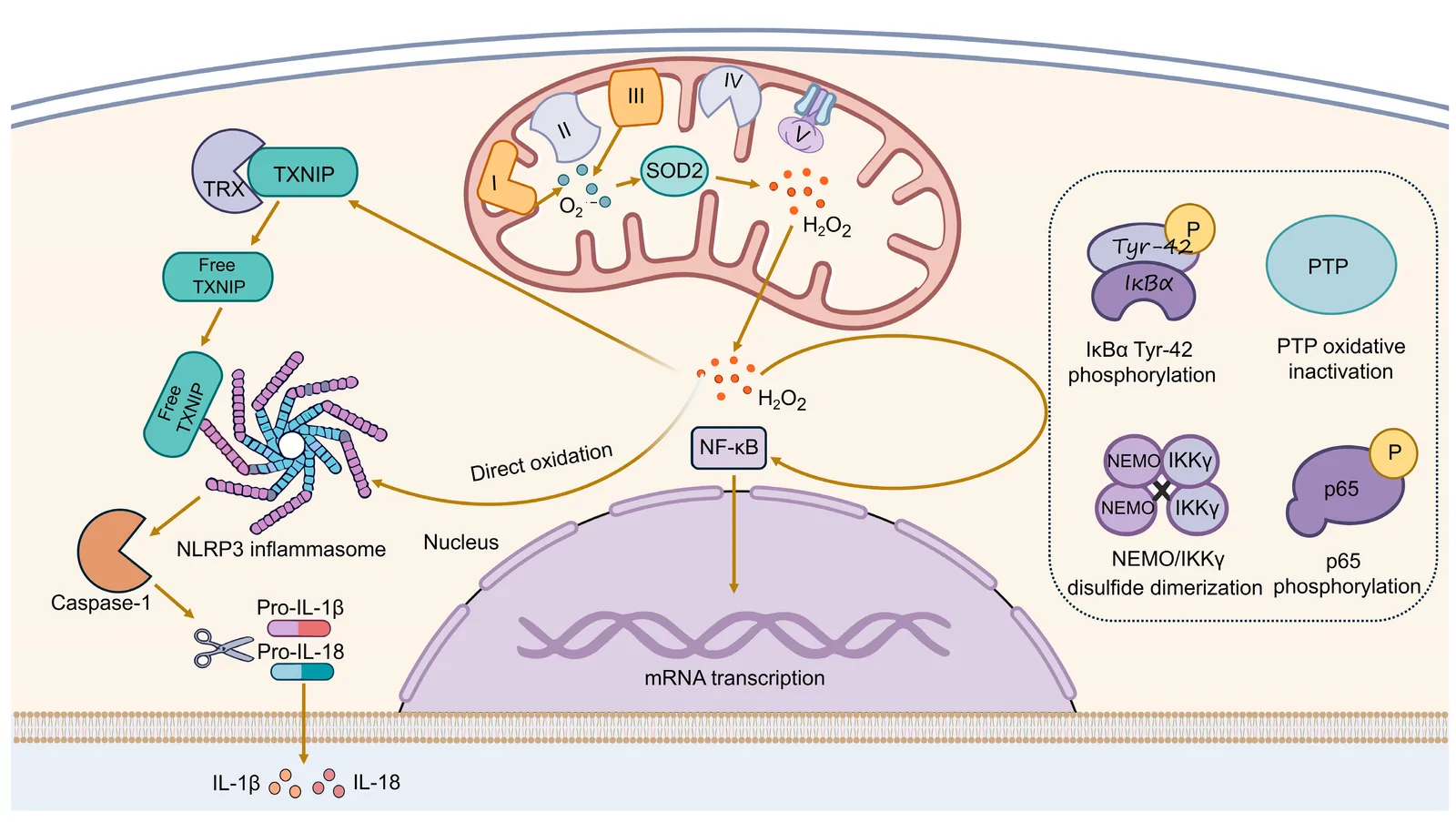
mtROS-mediated inflammatory signaling. Electron transport chain complexes I and III generate superoxide (O_2_·^−^), which is dismutated by matrix superoxide dismutase 2 (SOD2) to hydrogen peroxide (H_2_O_2_); H_2_O_2_ diffuses into the cytosol and activates two inflammatory arms. In the NLRP3 arm, H_2_O_2_ oxidizes thioredoxin (TRX) and releases bound thioredoxin-interacting protein (TXNIP); free TXNIP binds NLRP3 to nucleate inflammasome assembly, and H_2_O_2_ also engages NLRP3 by direct oxidation. In the NF-κB arm, H_2_O_2_ promotes NF-κB activation through four convergent routes: non-canonical IκBα Tyr-42 phosphorylation that bypasses the classical IKK pathway, oxidative inactivation of protein tyrosine phosphatases (PTPs), NEMO/IKKγ disulfide-bond-dependent dimerization, and p65 phosphorylation. Roman numerals denote respiratory chain complexes I to V; complexes I and III, the main superoxide-producing sites, are shaded orange. Arrows indicate the direction of signaling. Abbreviations: IκBα, inhibitor of NF-κB alpha; IKK, IκB kinase; IL, interleukin; NEMO, NF-κB essential modulator (also known as IKKγ); NF-κB, nuclear factor kappa B; NLRP3, NOD-like receptor family pyrin domain containing 3; p65, RelA subunit of NF-κB; P, phosphorylation.

Superoxide (O_2_·^−^) and hydrogen peroxide (H_2_O_2_) are produced at several sites in the mammalian electron transport chain, with complexes I and III the main contributors under most physiological conditions [49,50]. At these sites, leaked electrons reduce O_2_ to O_2_·^−^, which is subsequently dismutated by manganese superoxide dismutase (MnSOD/SOD2) in the mitochondrial matrix to H_2_O_2_. H_2_O_2_ is the principal ROS species capable of traversing mitochondrial membranes to function as a diffusible signaling molecule in the cytosol [49,51]. Under normal conditions, basal mtROS is maintained at a low level and participates in physiological signaling processes. However, when the electron leakage exceeds the scavenging capacity of the mitochondrial antioxidant defense system, pathological oxidative stress ensues [49,50,52]. Excess mtROS activates inflammatory signaling through multiple mechanisms (Figure 2).

First, mtROS modulates the NF-κB pathway through multiple indirect and highly cell-type-specific mechanisms. Contrary to earlier simplified models, Cys-179 of the inhibitor of NF-κB kinase subunit beta (IKKβ) is a target for oxidative inactivation by S-glutathionylation, which suppresses rather than activates IKK kinase activity and is reversed by glutaredoxin [53]. H_2_O_2_ instead acts through several indirect routes. The best characterized is phosphorylation of inhibitor of NF-κB alpha at Tyr-42, which proceeds independently of the classical Ser32 and Ser36 route: mutation of those serines blocks IL-1β-induced activation but leaves H_2_O_2_-induced activation intact, whereas Tyr-42 is required [54]. Oxidative inactivation of protein tyrosine phosphatases, disulfide-dependent dimerisation of NF-κB essential modulators, and ROS-dependent p65 phosphorylation contribute in parallel [55,56]. The net effect is that, during the early phase of inflammatory stress, mtROS generally promotes NF-κB activation in most types of immune cells, although sustained oxidative stress can paradoxically suppress NF-κB through proteasomal inhibition and cumulative oxidative inactivation of IKKβ [55,57]. Unlike ox-mtDNA-mediated NLRP3 activation, which depends on pre-existing priming signals within the cell, NF-κB activation upregulates the expression of both NLRP3 and pro-IL-1β and thereby supplies the priming signal for inflammasome activation [40]. A second mechanism involves the thioredoxin-interacting protein (TXNIP)–thioredoxin–NLRP3 relay. In resting cells, TXNIP is held in an inactive state by binding to thioredoxin. When mtROS levels rise, thioredoxin is oxidized, which forces TXNIP to release from thioredoxin. The freed TXNIP can bind NLRP3 directly and promote inflammasome assembly, and TXNIP deficiency impairs NLRP3 activation [58]. However, it should be noted that the structural basis of the TXNIP–NLRP3 interaction remains incompletely characterized, and mtROS likely activates the NLRP3 inflammasome through additional TXNIP-independent mechanisms, including direct oxidative modification of NLRP3 itself and potassium efflux-dependent pathways [59,60].

It is worth emphasizing that mtROS exhibits a characteristic “double-edged sword” feature. Moderate, transient elevations in mtROS (such as the low-level mtROS induced by exercise) do not cause cellular damage. Instead, they activate cytoprotective pathways, notably the Kelch-like ECH-associated protein 1 (Keap1)–nuclear factor erythroid 2-related factor 2 (Nrf2)–antioxidant response element (ARE) axis—whereby H_2_O_2_ oxidizes critical cysteine residues on Keap1 to release the transcription factor Nrf2 for nuclear translocation and transcriptional activation of antioxidant and cytoprotective genes—and the AMPK–PGC-1α–Sirtuin (SIRT) 1 axis—which senses energy stress and drives mitochondrial biogenesis and quality control [61,62,63]. These adaptive responses, together with mitochondrial unfolded protein response (UPRmt) and mitochondrial-derived peptides (e.g., humanin and MOTS-c) that function as systemic retrograde signals [62], collectively enhance the cell’s tolerance to subsequent stresses. This is the molecular basis of mitochondrial hormesis (mitohormesis) [61,62]. Only when mtROS generation chronically exceeds the cellular scavenging capacity does it transition from an adaptive signal to a pathological driver of inflammation [52,64]. This is the point at which exercise dose enters the argument. The mtROS pulse produced by a training bout normally sits within the adaptive range, whereas excessive or unaccustomed loads can push it past the clearance capacity of the cell, a dose dependence we return to below.

### 2.4. Other mtDAMPs and Multi-Pathway Synergistic Amplification

In addition to mtDNA and mtROS, other mtDAMPs contribute to the inflammatory network activated by mitochondrial dysfunction. Cardiolipin, a diphosphatidylglycerol phospholipid that accounts for roughly one-fifth of inner mitochondrial membrane phospholipids, stabilizes respiratory chain supercomplexes. Cardiolipin-deficient yeast still assemble complex III and IV supercomplexes, but these are markedly less stable, and reconstitution with cardiolipin largely restores the wild-type stability [65]. Upon mitochondrial damage, cardiolipin externalizes to the outer mitochondrial membrane surface. Knockdown of phospholipid scramblase 3 reduces this transport and decreases delivery of mitochondria to autophagosomes [66], and nucleoside diphosphate kinase D binds cardiolipin and facilitates its redistribution, with a binding-deficient R90D mutant losing the ability to promote mitophagy [67]. The process is therefore regulated rather than passive. Iyer et al. demonstrated that externalized cardiolipin directly binds NLRP3 to trigger inflammasome activation, and that interference with cardiolipin synthesis specifically inhibited NLRP3 assembly [68]. Notably, the immunological effects of externalized cardiolipin are modulated by its oxidation state: cytochrome c acts as a cardiolipin-specific oxygenase, catalyzing the oxidation of cardiolipin to generate oxidized cardiolipin, which is a prerequisite for the release of pro-apoptotic factors from the intermembrane space [69]. Moreover, oxidized cardiolipin, but not its native form, exhibits direct pro-inflammatory activity by inducing leukotriene B4 biosynthesis, elevating intracellular calcium, and upregulating adhesion molecules on endothelial cells [70]. This last point matters for the vessel wall since it places an additional mtDAMP alongside oxidized mtDNA in vascular inflammation. Mitochondrial N-formyl peptides, products of mitochondrial protein translation that retain the bacterial-type N-formylmethionine initiation, are recognized by formyl peptide receptor 1 on neutrophils once released from damaged mitochondria, and drive calcium flux, MAP kinase phosphorylation, migration, and degranulation [45]. Extracellular ATP released from damaged or dying cells activates the purinergic receptor P2X7, which lowers intracellular potassium [71]. A drop in cytosolic potassium is the common step shared by structurally unrelated NLRP3 agonists and is both necessary and sufficient for caspase-1 activation [72].

These diverse mtDAMPs, acting through distinct PRRs and signaling cascades, form an interconnected inflammatory network with extensive positive feedback and cross-amplification: mtROS promotes mtDNA oxidation; ox-mtDNA activates both NLRP3 and cGAS-STING; NLRP3-activated caspase-1 cleaves GSDMD, whose N-terminal fragment (GSDMD-NT) targets and permeabilizes both the IMM and the outer mitochondrial membrane (OMM) through binding to PLSCR3-externalized cardiolipin on the outer membrane—a process that occurs prior to plasma membrane damage and constitutes a “point of no return” for pyroptotic cell death—thereby releasing additional mtDAMPs, including mtDNA, mtROS, and mitochondrial proteins, from the matrix and the intermembrane space; STING engagement supplies a priming input for the inflammasome, traced in macrophages to type I interferon rather than to NF-κB [73]; secreted IL-1β further amplifies pro-inflammatory gene expression through IL-1R/MyD88/NF-κB signaling [41,47,74]. Two further observations close the loop. In macrophages, STING links mitochondrial fission to permeabilization through Drp1 and GSDMD-NT, enabling released mtDNA to re-engage cGAS-STING [75]; meanwhile, cytosolic ox-mtDNA binds GSDMD-NT and stabilizes the gasdermin oligomers [76]. Gasdermin pores therefore drive mtDNA release, and the released mtDNA reinforces the pores.

## 3. Exercise-Activated MQC: A Unified Mechanism for mtDAMP Suppression

**Figure 3:**
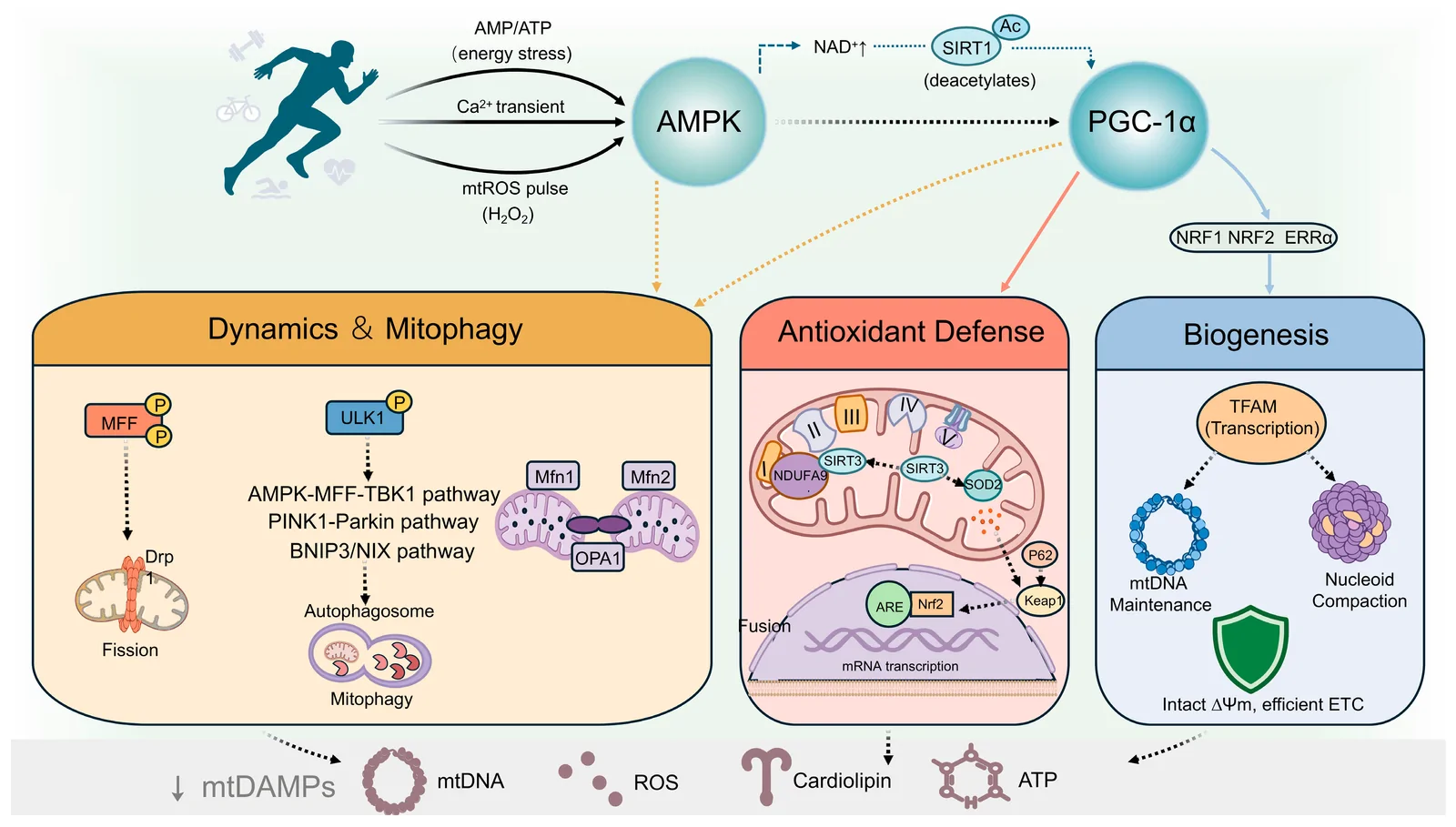
Exercise-activated mitochondrial quality control suppresses mtDAMP release. Exercise generates three signals (AMP/ATP shift, Ca^2+^ transient, mtROS pulse) that activate AMPK, which phosphorylates PGC-1α; SIRT1-mediated deacetylation then further increases its coactivator activity. The AMPK/PGC-1α axis coordinates four mitochondrial quality control (MQC) modules at complementary timescales. The integrated output is reduced release of mtDAMPs, including mtDNA, ROS, cardiolipin, and ATP. Solid arrows denote steps supported by evidence from human tissue, either in vivo or in isolated human cells; dashed arrows denote steps established only in animal models or in non-human cell systems. Arrow colors match the module each signal feeds: gold, dynamics and mitophagy; red, antioxidant defense; blue, biogenesis; black arrows are the exercise-derived inputs to AMPK and its output to PGC-1α, and the blue dotted route is the SIRT1 deacetylation branch. Roman numerals denote respiratory chain complexes I to V. Abbreviations: AMPK, AMP-activated protein kinase; ARE, antioxidant response element; BNIP3, BCL2/adenovirus E1B 19 kDa-interacting protein 3; Drp1, dynamin-related protein 1; ERRα, estrogen-related receptor alpha; ETC, electron transport chain; Keap1, Kelch-like ECH-associated protein 1; MFF, mitochondrial fission factor; Mfn1/2, mitofusin 1/2; mtDAMPs, mitochondrial damage-associated molecular patterns; NIX, NIP3-like protein X; Nrf2, nuclear factor erythroid 2-related factor 2; NRF1/2, nuclear respiratory factor 1/2; OPA1, optic atrophy protein 1; P, phosphorylation; p62, sequestosome-1/SQSTM1; PGC-1α, peroxisome proliferator-activated receptor gamma coactivator 1-alpha; PINK1, PTEN-induced kinase 1; SIRT1/3, sirtuin 1/3; SOD2, superoxide dismutase 2; TBK1, TANK-binding kinase 1; TFAM, mitochondrial transcription factor A; ULK1, Unc-51-like autophagy-activating kinase 1; Ψm, mitochondrial membrane potential.

It is well established that regular exercise exerts anti-inflammatory effects. These effects are mediated by multiple mechanisms, including reducing visceral fat mass, increasing the expression of anti-inflammatory proteins, reducing the circulating numbers of pro-inflammatory monocytes, and inactivating inflammatory signaling [77]. Although the relative importance of these different anti-inflammatory mechanisms remains unknown, more and more evidence highlights a critical role of MQC in exercise-afforded anti-inflammatory effects. This depends on the coordinated recruitment of four functional MQC modules, including biogenesis, antioxidant defense, dynamics, and mitophagy (Figure 3).

### 3.1. Terminology, Exercise Modality, and Dose

The literature reviewed below uses physical activity, exercise, and training as though the three were interchangeable, which hides distinctions that matter for the mechanism. We follow the established definitions: physical activity is any bodily movement produced by skeletal muscle that expends energy, exercise is the planned, structured, and repetitive subset undertaken to improve or maintain fitness, and physical fitness is the set of attributes that results [78]. Two further terms are used consistently here. A bout means one session, and training means repeated bouts over weeks to months. Unless a sentence states otherwise, exercise refers to training. The distinction is not pedantic in this context, because a single bout and a period of training move the same inflammatory markers in opposite directions, as noted for IL-6 in Section 1.

Intensity is reported as the source study defined it. Where a percentage is given, moderate intensity refers to 40 to 59% of heart rate reserve or oxygen uptake reserve and vigorous intensity to 60 to 89%, following the American College of Sports Medicine position stand [11]. Where a study reported only metabolic equivalents, absolute workload, or rating of perceived exertion, it is simply indicated rather than converted between scales, since these conversions are not equivalent in older adults or in patients with cardiovascular disease.

Two features of the dose recur in what follows. First, the relationship between training dose and mitochondrial quality is not monotonic. In healthy volunteers, a progressive increase in interval training volume raised mitochondrial content but impaired intrinsic mitochondrial respiratory function and reduced glucose tolerance at the highest volume, and both recovered when the load was reduced [79]. Mitochondrial content and mitochondrial function can therefore move in opposite directions once the load exceeds what the tissue can accommodate, which is the physiological basis of the hormesis problem discussed in Section 5.2. Second, the four modules of mitochondrial quality control are not recruited equally by every modality. Table 1 summarizes what each modality has been shown to engage, and in which tissue the evidence was obtained. An implication of this review is that no single prescription can be presented as optimal for all four modules, and the claims below are attached to a modality and an intensity wherever the primary literature allows.

The modalities are not interchangeable at the mitochondrial level. In human muscle, twelve weeks of resistance training raised coupled respiration without raising the transcripts that drive biogenesis [80], and six weeks of resistance training raised mitochondrial protein synthesis and respiration while leaving citrate synthase activity unchanged [81]. In a trial that compared high-intensity interval training, resistance training and the two combined in the same laboratory, mitochondrial respiration improved after interval and after combined training but not after resistance training alone [82], and only interval training increased mitochondrial volume and raised the fusion-to-fission protein ratio [83]. Resistance training therefore belongs here as a modality whose mitochondrial effects are real but narrower than those of endurance work, and one that is almost absent from the mtDAMP literature [84].

**Table 1:** Exercise modality, prescription, and the mitochondrial quality control modules for which direct evidence exists. A module is listed only where a study measured it, and the entries are restricted to the literature reviewed here rather than being a complete survey of the exercise physiology literature. MQC, mitochondrial quality control; mtROS, mitochondrial reactive oxygen species; Nrf2, nuclear factor erythroid 2-related factor 2; PGC-1α, peroxisome proliferator-activated receptor gamma coactivator 1-alpha.

Modality	Prescription as Defined in the Sources Cited	MQC Modules with Direct Supporting Evidence	Tissue from Which Evidence Was Obtained	Ref.
Moderate-intensity continuous training	40 to 59% of heart rate reserve or oxygen uptake reserve, 30 to 60 min, 3 to 5 days per week	Biogenesis; antioxidant defense	Human skeletal muscle biopsy; rat myocardium	[11,85,86,87,88]
High-intensity interval training	Repeated bouts at or above 90% of maximal heart rate separated by recovery periods	Biogenesis, with a transcriptional burst after each session; dynamics, shifted towards fusion; mitophagy	Human skeletal muscle biopsy; rat myocardium	[83,89,90,91]
Resistance training	60 to 80% of one-repetition maximum, 2 to 3 days per week	Respiratory function and mitochondrial protein synthesis rise without a matched increase in mitochondrial content; markers of mitochondrial dynamics unchanged in a head-to-head comparison with interval training	Human skeletal muscle biopsy; no cardiovascular data	[11,80,81,83,84]
Combined aerobic and resistance training	Both of the above within one program	Respiratory function improves to a similar extent as after interval training, while the shift towards fusion is smaller	Human skeletal muscle biopsy; clinical trials in cardiovascular populations	[82,83,92]
Single bout	One session of any of the above	Transient mtROS pulse, PGC-1α and mitofusin transcriptional bursts and acute Nrf2 activation; no measurable change in mitochondrial content	Human skeletal muscle biopsy	[89,93,94,95]

### 3.2. Convergent Sensing and Signal Integration

Three signals are biochemically early, muscle-wide, and sufficiently informative to trigger a coordinated transcriptional program during exercise: the ATP-to-AMP shift, the calcium transient, and the mtROS pulse generated by accelerated respiration [93]. AMPK satisfies the energy-sensing requirement. ATP hydrolysis during exercise raises the AMP/ATP and ADP/ATP ratios. AMP binding to the γ-subunit promotes Thr172 phosphorylation by liver kinase B1 (LKB1), protects Thr172 from dephosphorylation, and allosterically activates the phosphorylated enzyme, giving more than ten-fold activation in combination. ADP shares the protective effect but does not activate allosterically, so the two nucleotides are not interchangeable as signals [96]. Drake and colleagues demonstrated that a functionally distinct AMPK pool resides on the OMM and monitors organelle-level energetics with spatial specificity, complementing cytosolic AMPK signaling [97]. Calcium released from the sarcoplasmic reticulum activates calcium/calmodulin-dependent protein kinase kinase beta (CaMKKβ), which also phosphorylates AMPK Thr172 [98], and engages a distinct calcium/calmodulin-dependent protein kinase IV (CaMKIV)–calcineurin–myocyte enhancer factor 2 (MEF2) axis that drives PGC-1α transcription through an autoregulatory loop in which MEF2 binds the PGC-1α promoter and is coactivated by PGC-1α itself [99]. The contraction-associated mtROS pulse is sensed by two channels: H_2_O_2_ activates p38 mitogen-activated protein kinase (p38 MAPK), which directly phosphorylates PGC-1α at Thr262, Ser265, and Thr298 within the negative regulatory domain, thereby stabilizing the protein and enhancing its transcriptional activity [100]; and the same H_2_O_2_ signal oxidizes reactive cysteines on Keap1, releasing Nrf2 for nuclear translocation and ARE-driven antioxidant transcription [94]. These three sensing channels require a transcriptional integrator. Post-translational modification alone produces only minute- to hour-scale effects, whereas biogenesis, the expression of mitochondrial dynamics proteins, and antioxidant induction require sustained transcriptional remodeling. PGC-1α meets this requirement, receiving converging phosphorylation signals from AMPK at Thr177 and Ser538 [101] and from p38 MAPK [100], together with transcriptional signals from the CaMK–MEF2 axis [99], and coactivating multiple transcription factor families, including nuclear respiratory factor 1 (NRF1), nuclear respiratory factor 2 (NRF2), and estrogen-related receptor alpha (ERRα) that drive mitochondrial, antioxidant, and autophagy–lysosomal gene transcription [102]. Genetic evidence that p38γ-deficient mice fail to mount endurance exercise-induced mitochondrial biogenesis supports parallel rather than serial input [103].

Phosphorylation is necessary but not sufficient to activate PGC-1α fully. The coactivator is transcriptionally silent when its multiple lysine residues are acetylated; full release of its coactivator activity requires deacetylation by a NAD^+^-dependent enzyme [104]. This metabolic condition activates SIRT1, which deacetylates PGC-1α at multiple lysine residues and thereby increases its coactivator activity [104]. The AMPK–NAD^+^–SIRT1 axis therefore forms the second limb of PGC-1α activation, complementing AMPK-mediated phosphorylation. The sirtuin family logic extends across compartments. SIRT3 in the mitochondrial matrix performs analogous NAD^+^-dependent deacetylation of antioxidant and metabolic enzymes, considered separately in the antioxidant arm. SIRT6 in the nucleus interacts with NF-κB and deacetylates histone H3 lysine 9 at NF-κB target gene promoters, attenuating inflammatory gene expression and adding an epigenetic layer of restraint [105]. The AMPK–folliculin-interacting protein 1 (FNIP1)–transcription factor EB (TFEB)–PGC-1α cascade illustrates how the integrator network translates a single upstream pulse into a distributed transcriptional program. AMPK phosphorylates FNIP1 to release mechanistic target of rapamycin complex 1 (mTORC1)-mediated cytosolic sequestration of TFEB, which enters the nucleus and drives an early wave of lysosomal and autophagy genes, followed by a later PGC-1α/ERRα wave governing biogenesis [106]. One AMPK activation event thereby triggers both the clearance and synthesis machinery. Downstream, the integrated signal is distributed: NRF1, NRF2, and ERRα coordinate mitochondrial biogenesis; forkhead box O3a (FOXO3a) and the SOD2 promoter coordinate mitochondrial antioxidant defense; mitochondrial fission factor (MFF), mitofusin 1 (Mfn1), Mfn2, and optic atrophy protein 1 (OPA1) regulate mitochondrial dynamics; and Unc-51-like autophagy-activating kinase 1 (ULK1), TFEB, and BCL2/adenovirus E1B 19 kDa-interacting protein 3 (BNIP3) regulate mitophagy.

### 3.3. Mitochondrial Biogenesis: Reducing mtDAMP Generation at the Source

The most direct way to suppress mtDAMP release is to maintain a continuous cohort of newly synthesized mitochondria with intact membrane potential and efficient electron transport. The transcriptional program initiated in the sensing layer propagates through PGC-1α to NRF1, NRF2, and ERRα. Nuclear respiratory factor 1 binding sites in the human TFAM promoter are required for promoter activity, and mutating them reduces transcription [107]; expression of PGC-1 in muscle cells induces NRF-1 and NRF-2, and coactivation of NRF-1 raises TFAM expression and mtDNA content [108]. TFAM is the central executor of mitochondrial biogenesis because the mitochondrial genome occupies an unusual position: a 16.6 kb circular double-stranded genome encoding 13 essential respiratory chain subunits, yet lacking histones or equivalent structural protection. A single protein must therefore perform both the transcription/replication-initiating function and the structural packaging function. TFAM fills both roles.

Functionally, TFAM serves as the initiating factor of the mitochondrial RNA polymerase (POLRMT) complex, driving mtDNA transcription and replication, which in turn supports respiratory chain subunit synthesis [102]. Ekstrand and colleagues established a quantitative relationship using TFAM overexpression and heterozygous knockout mice: mtDNA copy number is directly proportional to total TFAM protein level, with heterozygous disruption reducing copy number by approximately 50% and general overexpression elevating it correspondingly [109]. Structurally, TFAM contains two high-mobility-group domains that each intercalate into the mtDNA minor groove, introducing a 180° U-turn that, combined with cooperative TFAM–TFAM contacts and cross-strand bridging, compacts the mtDNA from a ~5 μm contour length into a ~100 nm nucleoid [110,111,112]. The compacted, TFAM-coated state shields mtDNA from direct oxidative attack. TFAM heterozygosity promotes aberrant nucleoid packaging that triggers mtDNA escape into the cytosol and cGAS–STING-driven basal interferon signaling [113]. The stakes of preventing such escape are amplified by the recognition chemistry of cGAS itself: cGAS preferentially engages HMGB- and TFAM-bound U-turn DNA by forming protein-DNA ladders, so any TFAM-associated nucleoid fragment that reaches the cytosol generates more potent cGAS activation than naked mtDNA [114]. TFAM sufficiency therefore matters not because it makes escaped mtDNA less immunogenic but because it preserves nucleoid containment within intact organelles.

At the integrated physiological level, training elevates mtDNA copy number, mitochondrial volume density, citrate synthase activity, and ETC enzyme activities across species and training protocols [85]. Biopsy studies in human skeletal muscle have mapped the time course of this adaptation. In healthy young men who completed seven high-intensity interval sessions over two weeks, PGC-1α mRNA rose transiently after each bout and returned towards baseline within 24 h, while the protein content of nuclear-encoded and mitochondrially encoded respiratory chain subunits accumulated progressively across sessions. TFAM protein did not change over this period, which indicates that repeated transcriptional bursts precede any measurable change in the packaging protein itself [89]. Increases in mitochondrial content over longer training periods are summarized elsewhere [85]. Bishop and colleagues have recently emphasized that the magnitude and pattern of these adaptations depend on the prescription variables of type, intensity, frequency, and duration, since each variable engages the upstream sensors differently, and no single prescription can fully recruit all four MQC modules at once [90]. The biogenic response is not confined to contracting skeletal muscle. A multi-tissue, multi-omics survey of trained rats found coordinated mitochondrial remodeling across heart, liver, and adipose tissue, with PGC-1α-dependent gene programs forming a shared core, while each tissue showed distinct kinetics at the transcript, protein, phosphorylation, and acetylation levels [86]. Newly synthesized mitochondria with intact ΔΨm maintain restrictive outer and inner membranes, and TFAM-sufficient nucleoids resist the structural perturbation that drives mtDNA escape into the cytosol, where it activates cGAS-STING signaling [113]. Efficient ETC function lowers mtROS generation at the source [93]. Sufficient TFAM availability keeps mtDNA fully packaged; autophagy-compromised systems allow cytosolic mtDNA to accumulate and engage the NLRP3 inflammasome as signal 2 [7]. A single transcriptional program therefore attenuates three mtDAMP classes simultaneously.

### 3.4. Mitochondrial Antioxidant Defense: Intercepting mtROS Before It Becomes a Damage Signal

A bout of exercise increases respiratory flux, and mtROS production is therefore inevitable [93]. A dedicated antioxidant arm is required because mtROS occupies a triple role in the circuitry outlined earlier: as a direct DAMP engaging NF-κB-dependent inflammatory transcription, as the upstream generator of oxidized mtDNA (the nucleic-acid ligand for NLRP3 inflammasome activation), and as an indispensable signaling input for mitohormetic adaptation. The system must therefore handle mtROS with calibrated precision. Insufficient clearance permits inflammatory amplification, whereas blanket suppression abolishes adaptive signaling. Ristow and colleagues demonstrated the latter in humans: in men who completed four weeks of training, supplementation with vitamin C and vitamin E abolished the exercise-induced rise in superoxide dismutase and glutathione peroxidase expression in muscle and the accompanying improvement in insulin sensitivity, establishing that mtROS signaling is a required component of the adaptive response [87].

Two-layer architecture addresses this constraint. The mtROS generated within the matrix must be handled in situ; H_2_O_2_ that escapes to the cytosol must encounter a second tier with sufficient capacity to absorb the spillover while permitting its signaling function. The matrix layer centers on SIRT3. By the same NAD^+^-dependent logic that governs SIRT1 in the cytosol, an NAD^+^-sensitive deacetylase is required in the matrix to couple energy status to antioxidant output. SIRT3 deacetylates SOD2 to activate superoxide dismutase activity [115,116]. In parallel, SIRT3 physically associates with Complex I of the ETC and deacetylates the NDUFA9 subunit; SIRT3-null mice show hyperacetylated Complex I components and selectively impaired Complex I activity, establishing SIRT3 as a regulator of electron leak at the source [117]. The placement of SIRT3 within the AMPK–PGC-1α axis is supported by genetic evidence. In AMPKα2 kinase-inactive mice, exercise- and AICAR-induced upregulation of SIRT3 and SOD2 is abolished, and PGC-1α knockdown blocks the AICAR effect, placing SIRT3 downstream of the AMPK–PGC-1α axis [118]. The cytosolic layer is the Keap1–Nrf2–ARE pathway. H_2_O_2_ that diffuses across the outer mitochondrial membrane acts here not merely as a substrate but as an activating ligand: oxidation of reactive cysteines on Keap1 releases Nrf2 for nuclear translocation and binding to antioxidant response elements, driving transcription of antioxidant and detoxification genes [94]. In physically active men, a single bout of exercise to exhaustion raises Nrf2 Ser40 phosphorylation and total Nrf2 protein while lowering Keap1, with AMPK-dependent phosphorylation of p62/SQSTM1 at Ser349 targeting Keap1 for autophagic degradation; the elevated Nrf2/Keap1 ratio measured immediately after exhaustion is accompanied by a 2- to 3-fold increase in catalase protein [94]. This connects the Nrf2 arm directly to the AMPK network established in the sensing layer. FOXO3a further contributes to cytosolic MnSOD and catalase transcription, creating molecular overlap between the two antioxidant layers. The same H_2_O_2_ molecule that serves as a low-concentration Nrf2-activating signal is the substrate degraded at high concentration by catalase; local concentration thus dictates functional role.

The integrated stress signal that drives mitochondrial biogenesis through AMPK and PGC-1α also drives the antioxidant arm of training. In human muscle sampled at exhaustion, the rise in the Nrf2/Keap1 ratio is matched by increased catalase protein [94], which shows that the interception layer is inducible within a single session. Whether the trained state also carries a higher resting clearance capacity has not been tested with the same measurements, so the proposition that more H_2_O_2_ is intercepted at a given workload in trained than in sedentary muscle remains an inference [93]. Exercise therefore does not suppress mtROS generation; it raises the clearance set point so that mtROS occupies its physiological signaling range without spilling over into the inflammatory range. The antioxidant arm of MQC thus operates through the same upstream node, AMPK and PGC-1α together with their NAD^+^-dependent SIRT1 and SIRT3 cofactors, that governs the biogenesis and dynamics/mitophagy modules.

### 3.5. Mitochondrial Dynamics and Mitophagy: Isolating and Eliminating Damaged Mitochondria

When the first two arms operate optimally, the damage rate is minimized but not eliminated. Aging, acute high-intensity exercise, and pathological conditions still generate localized damage. Whether this residual damage triggers mtDAMP release depends on whether it can be segregated from the healthy network and cleared before membrane failure occurs. These two steps are physically coupled: imaging of exercised muscle in pMitoTimer reporter mice documents fission events that separate damaged mitochondrial segments prior to lysosomal engulfment [119]. Dynamics and mitophagy are therefore presented here as successive phases of a single process rather than as separate modules. AMPK directly phosphorylates MFF at Ser155 and Ser172 to recruit Drp1 and initiate fission within minutes [120]; PGC-1α transcriptionally upregulates nuclear-encoded mitochondrial genes, including mitochondrial dynamics components, on a timescale of hours to rebalance the network [102]. AMPK and PGC-1α therefore drive opposite phases of a single cycle rather than opposing pathways, and their temporal separation, rapid post-translational modification versus slower transcriptional remodeling, explains the sequential waves observed in vivo. Laker and colleagues mapped this sequence using pMitoTimer reporter mice: acute exercise triggered rapid Drp1 Ser616 phosphorylation, an oxidative stress peak between 3 and 12 h, and mitophagy flux peaking near 6 h [119]. The time course positions fission–mitophagy–fusion as three phases of a unified cycle.

The fusion phase of this cycle deserves explicit attention because it performs an anti-inflammatory function that complements fission and mitophagy rather than serving as a passive reset of network topology. Mfn1 and Mfn2, together with inner-membrane fusion executed by OPA1, enable content mixing between adjacent mitochondria to facilitate mitochondrial repair. Salvaging mildly damaged mitochondria by complementation lowers the number of organelles that must enter peripheral fission and mitophagy in the first place, which reduces the cumulative load on the clearance machinery. When fusion capacity falls behind fission rate, mildly damaged mitochondria accumulate as isolated fragments, lose membrane potential, and become vulnerable to the BAX/BAK- and VDAC-dependent permeabilization pathway that releases mtDAMPs into the cytosol [19,23]. The fusion arm is governed by the same PGC-1α-centered transcriptional program that drives biogenesis. Cartoni and colleagues found that a single bout of endurance exercise raised Mfn1 and Mfn2 mRNA in human skeletal muscle, with both transcripts peaking at 24 h post-exercise. Their reporter-gene assays in cellular systems demonstrated that Mfn2 transcription is driven by a PGC-1α-dependent program that requires ERRα, placing fusion within the same biogenic axis that drives respiratory chain expansion [95]. Chronic training reinforces this acute response. Twelve weeks of supervised aerobic training in obese older adults reduced mitochondrial fission protein 1 (FIS1) while preserving Mfn1, Mfn2, and OPA1, producing a higher fusion-to-fission protein ratio that tracked with improvements in glucose disposal [121]. In a cross-sectional comparison, habitually active men also had more fusion proteins in the mitochondrial-enriched fraction of skeletal muscle than sedentary men of the same age [122]. The arm is functionally necessary, not merely correlative. Adult-inducible skeletal-muscle-specific double knockout of Mfn1 and Mfn2 progressively reduces exercise capacity and ETC activity, whereas single knockout of either gene alone has no detectable phenotype, indicating that the two mitofusins act as a functionally redundant unit required for training adaptation [123]. The late fusion signal wave at 3 to 6 h after acute exercise documented by Laker and colleagues [119] reflects an active redistribution phase coordinated with biogenesis, fission, and mitophagy rather than a return to the resting baseline.

The transition from fission to mitophagy requires a selection signal to tag segregated damaged units for clearance. AMPK reappears at this step, phosphorylating ULK1 at Ser555 to initiate autophagy machinery assembly; Laker and colleagues demonstrated that ULK1 Ser555 phosphorylation is required for targeting of mitochondria to lysosomes in exercise-induced mitophagy [119]. Sustained severe energy stress can invert the AMPK–ULK1 relationship from activation to inhibition, which accounts for the apparent divergence between exercise responses and responses in chronic metabolic disease. Exercise operates within a dose window of transient, moderate-intensity pulses that fall within the activating range; chronic severe stress occupies a distinct, inhibitory regime. Three parallel receptor systems operate with distinct primary roles in mitophagy. The AMPK–MFF–TBK1 route, which functions independently of PTEN-induced kinase 1 (PINK1) and Parkin [124], predominates during acute exercise. The PINK1–Parkin canonical pathway handles baseline surveillance in oxidative fibers, activated by ΔΨm collapse through PINK1 stabilization on the outer membrane and subsequent Parkin-mediated ubiquitination [125], which subsequently recruits the autophagy receptors nuclear dot protein 52 (NDP52) and optineurin (OPTN) [126]. Receptor-mediated mitophagy through BNIP3 and NIP3-like protein X (NIX, also known as BNIP3L), established in developmental mitochondrial clearance during erythrocyte maturation [127] and in other tissues with high mitochondrial turnover demands, is extrapolated to slow-twitch skeletal muscle based on expression patterns and endurance training-induced upregulation, and this assignment should be treated as provisional until it is tested directly in that fiber type.

Loss-of-function evidence establishes the causal link between clearance failure and mtDAMP-driven inflammation. Autophagy inhibition in macrophages produces cytosolic mtDNA accumulation and NLRP3 inflammasome activation [7]; deoxyribonuclease II (DNase II) deficiency in cardiac tissue produces mtDNA retention in lysosomes and TLR9-driven sterile myocarditis and dilated cardiomyopathy [43]. Both models demonstrate that when mitophagy cannot keep pace with damage, mtDAMPs accumulate and engage innate immunity. The critical distinction from pathological circuits is the coupling partner of fission. Exercise-maintained fission is coupled to mitophagy, which delivers damaged segments to the autolysosomal system. In pathological states, cytosolic mtDNA pre-activates cGAS–STING [113], and a parallel pathway forms in which the N-terminal fragment of cleaved gasdermin D inserts into mitochondrial membranes, forming pores that release further mtDNA into the cytosol and amplifying cGAS–STING activation [128]. Each permeabilization event in this configuration becomes an occasion for additional mtDNA release, converting the dynamics module from a damage-resolution mechanism into a self-amplifying inflammatory circuit. Exercise-maintained MQC operates by keeping fission tethered to the mitophagy side of this fork, preventing entry into the pathological feedback loop.

The steady state achieved by trained muscle is not simply an elevated basal mitophagy flux but a combination of low baseline damage and a clearance machinery held in a primed state. This configuration is both preventive (biogenesis, antioxidant defense, and controlled dynamics reduce the damage load) and reactive (poised mitophagy machinery permits rapid clearance when damage does occur). The four MQC modules operate at complementary timescales: biogenesis on a slow, cumulative scale; antioxidant defense on an immediate interception scale; dynamics on a minute-scale segregation scale; and mitophagy on an intermediate-scale clearance scale. The threshold for inflammatory engagement is maintained by their conjunction, which defines MQC not as a set of independent housekeeping processes but as a coordinated mechanism for keeping mtDAMP release within a tolerated window.

## 4. Exercise–MQC–Inflammation Framework in Cardiovascular Disease and Aging

The framework developed in the preceding sections holds that exercise-derived signals converge on AMPK and PGC-1α pathways, drive coordinated activation of the four MQC modules, and thereby restrict mtDAMP release at its source. A claim of this generality cannot rest on internal logic alone. It must hold across pathological settings that differ in their dominant mtDAMP–PRR axis and in the complexity of the immune network engaged. Two complementary disease settings are needed to test the framework. The first type is a disease in which one mtDAMP–PRR pathway dominates; it tests whether exercise can shut down that pathway in a specific tissue compartment. The second type is a disease in which several pathways are engaged simultaneously and reinforce each other; it tests whether exercise can act at a network level rather than on any single pathway. The cardiovascular system supplies both within a single anatomy. One caveat applies to everything that follows. The mechanistic steps of this framework have been established almost entirely in rodent and cell models, whereas the human evidence is largely limited to changes in circulating inflammatory markers and in skeletal muscle mitochondrial content. We state the species and the model for each claim below, and Section 5.1 sets out what would be needed to close the gap. The myocardium operates almost entirely on oxidative phosphorylation, and its sterile inflammation is dominated by mtDNA–cGAS–STING signaling. The vasculature combines mechanical, redox, and metabolic stressors, and its sterile inflammation is dominated by ox-mtDNA–NLRP3 signaling. Cardiovascular aging engages both axes simultaneously, links them through NF-κB-mediated NLRP3 priming, and drives inflammaging as their joint output.

### 4.1. Cardiac Disease

The cardiovascular system is not metabolically homogeneous. Morphometric measurements of ventricular myocardium from ten mammalian species, including man, place the mitochondrial volume fraction at roughly a quarter to a third of cell volume [129], and cardiomyocytes depend almost entirely on oxidative phosphorylation for contractile work. A mitochondrial content this high means that even modest mtDNA damage can generate enough cytosolic fragments to engage cGAS. Vascular smooth muscle cells retain metabolic flexibility between glycolysis and oxidative metabolism, and endothelial cells operate under continuous laminar shear stress. In human endothelial cells, laminar shear stress promotes mitochondrial elongation, raises mitochondrial hydrogen peroxide production, and activates peroxiredoxin 3; hence, shear stress acts on both mitochondrial morphology and redox output [130]. Under this regime, fission-dominant dynamics and mtROS production become the prevailing stressors, and NLRP3, primed by NF-κB and activated by K^+^ efflux, mtROS, and oxidized mtDNA, becomes the dominant sensor. In the endothelium, PGC-1α connects these inputs to function: endothelial PGC-1α deletion lowers eNOS expression and impairs endothelium-dependent relaxation, whereas PGC-1α overexpression raises eNOS, which places mitochondrial biogenesis upstream of nitric oxide bioavailability [131]. Because the myocardium and vasculature channel mtDAMP signaling through different dominant PRR pathways, any unifying anti-inflammatory effect of exercise must act upstream of the point at which the two pathways diverge.

Cardiomyocytes occupy the first compartment of this dichotomy. Beyond its correlative elevation, cGAS–STING has been shown to contribute causally to cardiac pathology in several experimental settings, though the evidence does not cover every category of cardiac stress. In mice, cardiomyocyte-specific knockdown of cGAS rescued transverse aortic constriction-induced hypertrophy and dysfunction [132]. Subsequent work defined the upstream provocation: pressure overload induces inducible nitric oxide synthase (iNOS), which promotes cytosolic mtDNA release and activates the cGAS–STING–IRF3 cascade; in mice, cardiomyocyte-specific STING deletion or iNOS ablation each rescues the phenotype [133]. In a mouse model of ischemia and reperfusion injury, macrophage cGAS is required for the inflammatory component of myocardial damage after infarction [134]. In obese diabetic mice, lipotoxicity drives cytosolic mtDNA accumulation sufficient to activate cardiomyocyte cGAS–STING/IRF3/NF-κB signaling [135]. Closing the cGAS–STING axis pharmacologically is feasible through mitophagy enhancement. Augmented PARKIN-mediated mitophagy limits cytosolic mtDNA accumulation, suppresses cGAS–STING activation, and attenuates ischemia–reperfusion injury [136]. In mice, a small-molecule PARKIN activator, PR-364, enhanced mitophagy and mitochondrial biogenesis, preserved ejection fraction, and mitigated heart failure progression [137]. These studies establish that enhancing mitophagy is sufficient to suppress mtDAMP-driven sterile inflammation and post-infarct remodeling under active pathological insult. Exercise meets this criterion at the AMPK–ULK1 node. In mice, the genetic work of Laker and colleagues established that ULK1-Ser555 phosphorylation by AMPK is required for exercise-induced mitophagy, providing the molecular link between contractile signaling and damaged-mitochondria clearance [119]. In rats given four weeks of interval treadmill training after coronary ligation, myocardial microtubule-associated protein 1 light chain 3, FUN14 domain-containing protein 1, PINK1, and Parkin all rose. The same report also contains a separate trial in which twelve weeks of exercise-based cardiac rehabilitation raised left ventricular ejection fraction in patients after myocardial infarction, with no mitophagy marker measured in that arm [91]. The irisin/fibronectin type III domain-containing protein 5 axis provides an exerkine-mediated route: exercise-induced circulating irisin activates cardiac PINK1/Parkin signaling and improves post-infarct cardiac function [138]. Mitophagy-mediated cardioprotection is therefore not exclusive to exercise but represents a shared endpoint reachable through several physiological perturbations, of which exercise is the safest and most integrative. Two qualifications apply to the causal evidence for this axis. It rests on genetically modified mice; what has been shown is that cGAS–STING is necessary in those models, not that it is necessary in the human heart. The phenotype also depends on which cell carries the deletion, since the studies above remove cGAS or STING from cardiomyocytes in some cases and from macrophages in others, and the protection obtained is not interchangeable between the two. The axis is therefore better read as context-dependent than as a universal requirement for adverse remodeling.

Direct human evidence for this step is thinner than the preclinical evidence, and it is worth stating where it stops. In patients with heart failure with preserved ejection fraction, a randomized trial of supervised endurance and resistance training improved peak oxygen uptake and diastolic function over three months [92]. The trial did not measure mtDNA release, cGAS-STING activity, or myocardial mitophagy, so it establishes that training changes the clinical phenotype this framework is meant to explain, not that it does so through the proposed mechanism. No human study has yet connected a training program to a change in myocardial mtDAMP handling. Section 5.1 treats this as the central translational gap.

### 4.2. Vascular Disease

In atherogenesis, oxidized low-density lipoprotein (LDL) is bound by lectin-like oxidized LDL receptor 1 (LOX-1), an endothelial receptor identified by expression cloning from vascular endothelial cells [139]. Binding of oxidized LDL to LOX-1 lowers intracellular nitric oxide in bovine aortic endothelial cells by increasing superoxide production, and an antibody against LOX-1 prevents the superoxide rise [140], which places impaired nitric oxide bioavailability rather than frank injury at the proximal end of the sequence. The resulting oxidized mtDNA is generated through a cytidine/uridine monophosphate kinase 2 (CMPK2)-dependent pathway that is required for full NLRP3 inflammasome assembly in macrophages [9]. CMPK2 thus serves as the enzymatic gatekeeper for ox-mtDNA formation. The cellular locus of NLRP3-driven vascular inflammation extends beyond macrophages: in human coronary atherosclerosis, immunohistochemical analysis attributed approximately 50% of intimal foam cells to a smooth muscle cell origin rather than a myeloid one [141], revising the classical macrophage-centered narrative and placing NLRP3 activation in VSMC transdifferentiation at the center of plaque biology. Human causality has been independently secured through the Canakinumab Anti-inflammatory Thrombosis Outcomes Study (CANTOS), in which monoclonal antibody neutralization of IL-1β reduced recurrent cardiovascular events without lipid modification [142], providing the only randomized-trial-level confirmation that the NLRP3/IL-1β axis is pathogenic in human atherosclerosis. Three lines of evidence, enzymatic, cellular, and clinical, converge on ox-mtDNA–NLRP3 as the dominant vascular axis, in sharp contrast to the cGAS–STING dominance established for the myocardium.

Training engages the vasculature through several mechanistic layers, including biogenesis, dynamics, antioxidant defense, epigenetic regulation, and inter-organ communication. Laminar shear stress activates the canonical AMPK–SIRT1–PGC-1α–NRF1–TFAM cascade, increasing endothelial mtDNA copy number and respiratory capacity [143]. Reviews of vascular mitochondrial biology place PGC-1α at the point where mechanical stress, redox signaling, and quality control converge [144]. Swimming training in spontaneously hypertensive rats suppressed Drp1 expression and restored fission–fusion balance in the aortic endothelium; in the human arm of the same study, mesenteric artery endothelium from patients with hypertension showed the same fragmentation and the same Drp1 elevation, although the effect of exercise was tested only in the rats [145]; irisin suppressed Drp1 expression in calcifying vasculature [146]. Epigenetic regulation provides a direct molecular bridge to NLRP3. In mice, Yang and colleagues showed that exercise downregulates methyltransferase-like 14 (METTL14)-mediated N6-methyladenosine (m^6^A) modification of the long non-coding RNA nuclear enriched abundant transcript 1 (NEAT1), which destabilizes NEAT1 and reduces its scaffolding of Krüppel-like factor 4 (KLF4), thereby attenuating KLF4-driven NLRP3 transcription and endothelial pyroptosis [147]. The inter-organ layer is illustrated by Lou and colleagues: exercise induced liver-derived extracellular vesicles, which enhanced endothelial fatty-acid utilization and promoted angiogenesis [148]. Myocardium and vasculature thus channel inflammation through different dominant PRR pathways but share an upstream MQC-level intervention point.

Human evidence here has a similar shape. In patients with chronic heart failure, six months of supervised endurance training lowered local expression of tumor necrosis factor alpha, interleukin-1β and interleukin-6 in skeletal muscle, while the circulating concentrations of the same cytokines changed little [149]. Two things follow. Training can lower inflammatory signaling in the tissue where mitochondrial adaptation occurs, and the circulating concentration is a poor reporter of that local change. The second point is one reason for proposing mtDAMP release rather than circulating cytokine concentrations as the readout of this mechanism, an argument taken up in Section 5.1. The note on timing applies to this section as a whole. Atherosclerosis in humans develops over decades, and the work cited here samples stages of that process rather than discrete events. Where the text contrasts one setting with another, the contrast is between early lesion formation and established plaque, not between acute and chronic disease.

### 4.3. Cardiovascular Aging

Cardiovascular aging poses a more demanding test, for three reasons that lie outside the compartment-specific dichotomy considered above. First, cGAS–STING and NLRP3 activate concurrently and reinforce each other through NF-κB-mediated NLRP3 priming, a crosstalk that is dispensable in acute pathology but functionally decisive in chronic accumulation [150]. Second, the two axes share a common upstream driver, NAD^+^ depletion with coordinated SIRT1/SIRT3 inactivation, whose systemic decline is a defining feature of aging [151,152]. Third, the temporal balance between mitochondrial-damage generation and clearance acquires decisive weight only over a decade-scale process [153]. Clinically, cardiovascular aging manifests as heart failure, arterial stiffening, endothelial dysfunction with reduced flow-mediated dilation, and inflammaging as their integrated molecular signature [154].

Among the two axes, cGAS–STING has accumulated the strongest causal evidence in aging pathology. Victorelli and colleagues demonstrated that senescent cells release mtDNA through minority mitochondrial outer-membrane permeabilization, and that this cytosolic mtDNA drives the senescence-associated secretory phenotype (SASP) through cGAS–STING-dependent inflammatory signaling [155]; pharmacological blockade of BAX/BAK pore formation attenuated SASP, establishing that the age-associated inflammatory phenotype can be reversed by closing mtDAMP release at its source. Within the vasculature, telomere damage in vascular smooth muscle cells is associated with cytoplasmic DNA accumulation and with cGAS-STING-dependent inflammatory signaling during senescence [156]. How this relates to the NLRP3-dominant picture described above for atherogenesis has not been tested directly. The two observations are compatible if the relative contribution of the two sensors shifts with the duration of the insult, but that ordering is an inference drawn across separate studies rather than a measured result. Recent studies suggest cGAS–STING activation as a functionally significant contributor to multiple cardiovascular diseases [157]. Crosstalk between the two axes is the single feature that most sharply distinguishes cardiovascular aging from acute cardiovascular disease.

The direction of this coupling needs to be stated carefully, because the two axes do not simply reinforce one another. In mouse macrophages, cGAMP primes the NLRP3 inflammasome through a STING- and type I interferon-dependent route rather than through NF-κB, and it also promotes lysosomal cell death that supplies the second signal [73]. Type I interferon acts in the opposite direction on the same axis, repressing pro-IL-1β transcription and limiting inflammasome-dependent IL-1β maturation [158]. Whether chronic STING activation is on balance amplifying or restraining for NLRP3 therefore depends on the duration and magnitude of interferon signaling, and this has not been resolved. What can be said is that both axes are engaged concurrently in aged cardiovascular tissue and that both lie downstream of the same mtDAMP load. In acute or subacute settings, one pathway dominates so decisively that cross-pathway amplification is dispensable; only in chronic, cumulative aging does this crosstalk become functionally decisive, generating the self-sustaining inflammatory spiral. NLRP3 retains an independent pathogenic role in the aged myocardium. In heart failure, persistent NLRP3 inflammasome and IL-1β signaling have been causally linked to interstitial fibrosis and diastolic stiffening [159]. Concurrent activation of the two inflammatory axes demands a common upstream driver, and this driver has been identified at the cofactor level. NAD^+^ declines progressively with age, and because SIRT1 and SIRT3 both require NAD^+^ as an obligate cofactor, their activities collapse in parallel, disabling deacetylation of PGC-1α, SOD2, and FOXO3a and dismantling all four MQC modules simultaneously [151,152].

The integrative nature of cardiovascular aging pathology dictates that any effective intervention must act at a network level rather than on any single pathway, and the evidence for exercise meeting this criterion operates across four registers. At the cellular level, Rossman and colleagues found that p53, p21, and p16 expression in endothelial cells of habitually active older adults is indistinguishable from that of young sedentary controls, with flow-mediated dilation correspondingly preserved [160]. At the mitophagy-machinery level, older active adults retain markedly elevated PARKIN abundance in the mitochondrial fraction of skeletal muscle relative to inactive peers [122], and mitophagy markers rise in the myocardium of rats trained after coronary ligation, in the rodent arm of the study described in Section 4.1 [91]. Temporal dynamics supply a complementary mechanism. Jiménez-Loygorri and colleagues demonstrated that when mitophagic capacity is insufficient to match the rate of mitochondrial damage, cytosolic mtDNA accumulates progressively and engages cGAS-STING, driving a type I interferon response that scales with the degree of mitophagy impairment [161]. Exercise-induced mitophagy may thus restrain this inflammatory cascade by maintaining clearance efficiency above the threshold at which mtDNA escapes into the cytosol.

**Figure 4:**
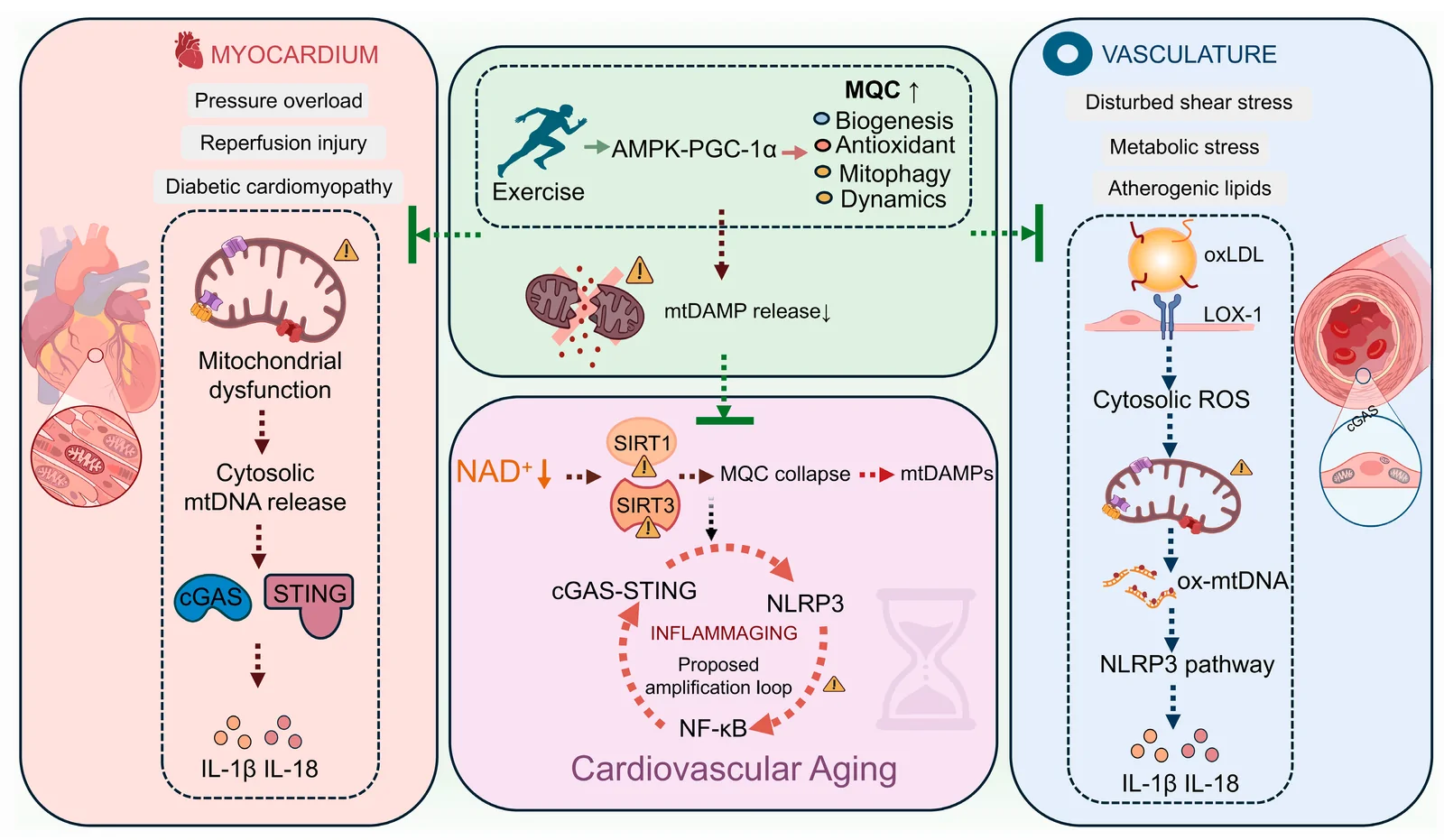
Exercise-activated MQC suppresses mtDAMP-driven inflammation in the cardiovascular system. Cardiovascular sterile inflammation runs through compartment-specific dominant axes. In the myocardium, pressure overload, ischemia/reperfusion injury, and metabolic disorder release intact mtDNA nucleoids from cardiomyocyte mitochondria, activating cGAS-STING signaling and driving IL-1β and IL-18 production. In the vasculature, oxidized low-density lipoprotein (oxLDL) engages LOX-1 on endothelial cells to trigger CMPK2-dependent ox-mtDNA generation, which activates the NLRP3 inflammasome and produces IL-1β and IL-18. In cardiovascular aging, progressive NAD^+^ depletion inactivates SIRT1 and SIRT3 in parallel, dismantling the four MQC modules and elevating mtDAMP load; cGAS-STING and NLRP3 are then engaged concurrently, and the arrows linking them are drawn dashed because the direction of this coupling is not resolved (Section 4.3). Exercise is proposed to restrain all three axes upstream of the pathway divergence through enhanced mitophagy, biogenesis, dynamics, and antioxidant defense. Solid arrows denote steps supported by evidence from human tissue, either in vivo or in isolated human cells; dashed arrows denote steps established only in animal models or in non-human cell systems. Panel colors identify the compartments: red, myocardium; blue, vasculature; purple, cardiovascular aging; green, the exercise-activated MQC response, and arrows take the color of the compartment they belong to. Green bar-headed lines denote inhibition, and warning symbols mark sites of mitochondrial damage or loss of function. Abbreviations: cGAS, cyclic GMP-AMP synthase; IL, interleukin; LOX-1, lectin-like oxidized LDL receptor 1; MQC, mitochondrial quality control; mtDAMPs, mitochondrial damage-associated molecular patterns; mtDNA, mitochondrial DNA; NAD^+^, nicotinamide adenine dinucleotide; NF-κB, nuclear factor kappa B; NLRP3, NOD-like receptor family pyrin domain containing 3; ox-mtDNA, oxidized mtDNA; SIRT1/3, sirtuin 1/3; STING, stimulator of interferon genes.

Across the single-pathway-dominant settings of cardiovascular disease and the multi-pathway-convergent setting of cardiovascular aging, one mechanistic claim holds: myocardial cGAS–STING, vascular NLRP3, and their chronic crosstalk are all suppressed upstream of pathway divergence, at the level of MQC-mediated mtDAMP restraint (Figure 4). The practical significance lies in this upstream locus. Pharmacological interventions that block downstream effectors illustrate this trade-off. Canakinumab, which neutralizes IL-1β in the CANTOS trial, is a classic example [142]. It reduced cardiovascular events but was associated with a higher incidence of fatal infection [142]. Training is not subject to this particular trade-off since it does not neutralize circulating cytokines. Whether it would produce a comparable reduction in events through the upstream route proposed here has not been tested, and the comparison is offered as a hypothesis rather than as a demonstrated advantage.

### 4.4. Clinical Considerations

This framework will be tested, if at all, in the conditions clinicians see most often, and the human evidence is not equally strong across them. In hypertension, a meta-analysis of 93 randomized trials in 5223 adults found that endurance, dynamic resistance and isometric training each lower resting blood pressure [162], whereas the mitochondrial part of that effect has so far been shown only in animals, in the swimming study in spontaneously hypertensive rats described in Section 4.2 [145]. In type 2 diabetes and obesity-related cardiomyopathy, cytosolic mtDNA accumulation sufficient to activate cardiomyocyte cGAS-STING has been demonstrated in obese diabetic mice [135], while the human trials reporting improved skeletal muscle mitochondrial outcomes are mostly pre-post rather than randomized [163]. In heart failure with preserved ejection fraction, supervised training improved peak oxygen uptake and diastolic function in a randomized trial [92], with no mtDAMP endpoint measured. After myocardial infarction, exercise-based cardiac rehabilitation raises left ventricular ejection fraction in patients, while the mitophagy markers set out in Section 4.1 come from the rodent arm of the same study [91]. The pattern is the same in all four settings: the clinical benefit is established, the mitochondrial mechanism is inferred, and we have left that gap visible rather than writing over it.

One population deserves separate mention, because it is the one in which the usual anti-inflammatory options are least available. Solid organ transplant recipients carry a high inflammatory and oxidative burden, and pharmacological suppression of inflammation is constrained by the immunosuppression they already receive and by the infection risk that comes with it. An intervention acting upstream of pattern recognition receptor engagement is attractive in that setting for exactly this reason, since it does not add to systemic immune suppression. Exercise is feasible in these patients: in a multicenter randomized trial, 81 heart transplant recipients started training a mean of eleven weeks after transplantation and completed nine months of either high-intensity interval training or moderate-intensity continuous training, with peak oxygen uptake as the primary outcome [164]. Whether training also lowers mtDAMP release in this group has not been tested, and the transplanted heart is denervated, so its response to a training stimulus cannot be assumed to match that of an innervated one. We raise the setting as the place where the framework would matter most if it holds, not as a place where it has been shown to hold.

Two practical obstacles stand between this framework and the clinic. The first is measurement. Mitochondrial quality control is not part of any routine laboratory panel, and no mtDAMP is a validated clinical analyte. Circulating cell-free mtDNA, the one most often measured, is sensitive to centrifugation and extraction conditions, varies with the target gene chosen for amplification, and sits near the limit of quantification in healthy people at rest, as set out in Section 5.1. Three things would have to be in place before a clinician could act on such a measurement: a pre-analytical protocol agreed across laboratories, a reference interval established in the relevant age and disease groups, and evidence that a change in the analyte tracks a change in outcome rather than merely accompanying it. None of the three exists yet. The second obstacle is adherence. Participation in and completion of cardiac rehabilitation fall well short of what the programs are designed to deliver [165], and the methodological consequence is easy to overlook: when adherence is partial, the delivered dose is lower than the prescribed dose, so analysis by prescribed dose biases any association between exercise and mtDAMP release toward the null. Trials that set out to test this framework should measure the delivered dose objectively and analyze outcomes by that measured dose.

## 5. Perspectives

### 5.1. Translational Gaps and Biomarker Limitations

The mechanistic evidence underpinning the framework derives predominantly from rodent and cell culture models. Cellular and animal models differ from human physiology in metabolic rate, fiber-type composition, and mitochondrial regulation, and these differences complicate direct extrapolation [166]. Rodent exercise models also introduce specific confounders. Campeau et al. demonstrated that six weeks of voluntary wheel running in rats reduces hypothalamic–pituitary–adrenal (HPA) axis response to low-intensity stressors, while noting that forced treadmill paradigms in the literature are associated with chronic HPA activation and adrenal hypertrophy [167]. This distinction matters because the stress milieu created by the exercise model fundamentally alters the context in which mitochondrial adaptations occur. In human exercise trials, the evidence is further limited by study design. Most trials measure either upstream MQC markers—such as citrate synthase activity and PGC-1α protein—or downstream inflammatory endpoints—such as CRP, IL-6, and TNF-α—but omit the intervening mtDAMP step. A recent systematic review and meta-analysis of exercise effects on skeletal muscle mitochondrial outcomes in type 2 diabetes reported that the majority of included studies used pre-post designs rather than randomized controlled trials [163]. Without simultaneous measurement of MQC, mtDAMPs, and inflammatory pathway activation in the same cohort, the causal links between these levels of the framework remain inferred rather than directly demonstrated. We searched specifically for a human exercise trial that measured a marker of mitochondrial quality control together with an mtDAMP or an innate immune readout in the same participants and did not find one. The closest is the chronic heart failure trial described in Section 4.2, in which training lowered local inflammatory gene expression in skeletal muscle, but that trial measured no mtDAMP. The central link of this framework, from improved mitochondrial quality control to reduced mtDAMP release, therefore has no direct human support at present. This is the largest single gap in the argument, and we state it here rather than leave it to be inferred.

Biomarker limitations further constrain this effort. Circulating cell-free mtDNA (ccf-mtDNA), the most frequently measured mtDAMP in exercise studies, is sensitive to pre-analytical variables, including centrifugation speed, DNA extraction method, and sample type. Daubermann et al. demonstrated that ccf-mtDNA concentrations are far more sensitive to centrifugation force than nuclear cell-free DNA, highlighting the need for standardized pre-analytical protocols [168]. Target gene selection for quantitative PCR varies across laboratories, and because mtDNA fragmentation is region-specific, with hotspots in the RNR2 and COX2 regions documented in sepsis patients [169], this inconsistency affects cross-study comparability. In healthy individuals at rest, ccf-mtDNA concentrations approach the lower limit of quantification [168], so exercise-induced changes in non-diseased populations may be too small to measure reliably with standard assays. Nuclear mitochondrial pseudogenes, numbering in the hundreds in the human genome, introduce contamination risk that grows as true mtDNA concentration falls [170]. For mtROS, cardiolipin, TFAM, N-formyl peptides, and extracellular ATP, the measurement challenges are greater: none of these mtDAMPs have been quantified systematically in exercising human populations. Developing standardized mtDAMP panels is an important methodological need for advancing this field.

Table 2 sets out where each step of the framework stands. The grading is deliberately coarse. A step counts as human evidence only when it was measured in human participants in vivo, as animal evidence when it rests on work in intact animals, and as in vitro evidence when it rests on isolated cells or tissue. Figure 3 and Figure 4 carry a coarser two-way form of the same distinction, with solid arrows for steps supported by evidence from human tissue and dashed arrows for steps established only in animal models or in non-human cell systems. Where a single study reports both a human and an animal arm, the text names the arm each statement comes from. Reading down the third column makes the shape of the problem plain: the upstream half of the sequence is supported in humans, while the downstream half is not.

### 5.2. The Hormesis Paradox and Unresolved Prescription Questions

The mitohormesis model posits that moderate mitochondrial stress during each exercise bout activates Nrf2-mediated antioxidant defense, AMPK-driven biogenesis, UPRmt, and selective mitophagy, and that repeated engagement of these pathways gradually raises mitochondrial quality and reduces resting mtDAMP burden [171,172]. Several aspects of this model, however, remain untested. The cellular origin of acutely released ccf-mtDNA is unclear: passive leakage from damaged cells and regulated secretion through mitochondria-derived vesicles would carry different biological meanings, but no method currently distinguishes between the two in the exercise context. The boundary between hormetic stress and outright mitochondrial damage has not been mapped with precision. Flockhart et al. found that four weeks of progressively increasing high-intensity interval training led to impaired mitochondrial respiration and glucose intolerance at peak load; a subsequent recovery week partially restored these parameters, but intrinsic mitochondrial respiration did not fully return to moderate-training levels within that timeframe [79]. This observation suggests that the anti-inflammatory predictions of the present framework only hold within a specific dose range.

The absence of a dose–response map linking exercise prescription parameters to MQC outcomes and mtDAMP suppression limits practical application. A meta-regression encompassing 353 studies and over 5600 participants found that total training load predicted mitochondrial content changes, and that higher training frequencies were associated with larger increases in citrate synthase activity in a positive, roughly linear relationship [88]. Sprint interval exercise and moderate-intensity continuous training activate different branches of MQC: the former triggers the integrated stress response and UPRmt, while the latter preferentially increases respiratory chain content and coupling efficiency [172]. Whether these distinct molecular responses produce different patterns of mtDAMP release has not been investigated, and resistance training is almost entirely absent from the mtDAMP literature [84]. Population heterogeneity adds another layer of complexity. Women show higher intrinsic mitochondrial respiration per unit mitochondrial protein than fitness-matched men [173], and estrogen regulates mitochondrial biogenesis through NRF-1 and TFAM [174], yet no study has tested whether menopause or menstrual cycle phase modulates exercise-induced mtDAMP release. In older adults, reduced AMPK and SIRT1 activity and impaired autophagic flux may diminish the MQC response to a given exercise stimulus [166], raising the possibility that older individuals accumulate more, not fewer, mtDAMPs from the same training program. Longitudinal studies that track mtDAMP kinetics across repeated exercise bouts, stratified by modality, dose, sex, age, and disease status, are needed to address these gaps.

### 5.3. MDVs and Exercise Mimetics

Among the research directions that could advance this framework, three are particularly promising. MDVs represent a layer of MQC that acts before canonical mitophagy. König and McBride reviewed evidence that MDV generation increases within two to six hours of mild stress, well ahead of the twelve to twenty-four hours required for mitophagy activation, and that MDVs selectively remove damaged mitochondrial components without disposing of entire organelles [175]. Todkar et al. showed that Parkin-dependent MDVs deliver damaged mitochondrial content to lysosomes, while sorting nexin 9-dependent MDVs package healthy mitochondrial proteins into extracellular vesicles for intercellular signaling [176]. When Parkin was deleted, pro-inflammatory mitochondrial content was no longer sorted to lysosomes but instead exported via extracellular vesicles, suggesting that MDV cargo selection directly determines whether intercellular communication is reparative or inflammatory. Exercise is known to increase circulating extracellular vesicle concentrations, but whether exercise alters MDV biogenesis, sorting fidelity, or mitochondrial cargo composition is entirely unknown.

Pharmacological MQC enhancers offer a complementary strategy for individuals unable to exercise at sufficient intensity. Urolithin A, a gut microbiota-derived mitophagy inducer, improved hamstring muscle strength and aerobic endurance while reducing plasma CRP in a four-month randomized trial of middle-aged adults, although the primary endpoint of peak power output was not met [177]. A subsequent placebo-controlled trial showed that 28 days of urolithin A supplementation expanded naive CD8^+^ T cells and improved monocyte phagocytic function in adults [178]. These results support the idea that pharmacological mitophagy induction can produce anti-inflammatory effects, but no trial has yet measured whether urolithin A or any other exercise mimetic reduces mtDAMP concentrations in humans. Demonstrating this link would provide a direct pharmacological test of the framework’s central prediction. Because exercise activates multiple MQC branches simultaneously across tissues while individual compounds typically engage only one, combining exercise with pharmacological MQC support may prove more effective than either approach alone, but such combination trials have not been conducted.

### 5.4. Future Directions

The gaps set out above point to work of three different kinds.

For basic research, what is needed is a conditional test of necessity rather than another correlation. The framework predicts that blocking the mitochondrial quality control response to exercise should abolish the reduction in mtDAMP release while leaving the contractile response intact. Inducible, tissue-restricted deletion of the components that carry the signal, combined with reporters that read mtDNA in the cytosol rather than in the circulation, would provide that test. The same design would settle whether the cardiac and vascular effects require a local response or can be conferred by circulating factors released from working muscle, a question this review has had to leave open.

For clinical research, the immediate need is measurements rather than interventions. Before a trial can test the central claim, it requires an mtDAMP panel that behaves reproducibly, which means a pre-analytical protocol agreed across laboratories, a target region chosen with mtDNA fragmentation in mind, and reference intervals established for the concerned age groups. Once that exists, the study that would test the framework most directly is a dose-finding trial in middle-aged and older adults with cardiovascular disease, measuring mitochondrial quality control markers and mtDAMP concentrations in the same participants at the same time points, and using delivered rather than prescribed dose as the exposure variable.

For exercise instruction and prescription, the practical consequence follows only if mtDAMP load turns out to be the better target. If it does, the prescription would be titrated to a measured biological response rather than to a percentage of maximum, and the non-monotonic dose response described in Section 3.1 would become a reason to monitor rather than a caveat to mention. Until then, the honest position is that existing recommendations, which rest on cardiorespiratory and clinical endpoints, remain the ones to follow, and that this framework changes what should be measured in the next generation of trials rather than what should be prescribed today.

## 6. Conclusions

This review argues that exercise reduces chronic inflammation by improving mitochondrial quality, with mtDAMPs serving as the mechanistic link between mitochondrial dysfunction and innate immune activation. When mitochondrial integrity is lost, mtDNA, mtROS, cardiolipin, N-formyl peptides, and ATP escape into the cytosol or extracellular space and activate cGAS–STING, NLRP3, TLR9, AIM2, ZBP1, and NF-κB signaling. Crosstalk and positive feedback among these pathways allow even mild mitochondrial damage to develop into persistent low-grade inflammation. Regular exercise opposes this cascade through the AMPK-PGC-1α axis, which coordinates four MQC arms: biogenesis, antioxidant defense, dynamics, and mitophagy. These arms act on different time scales and together hold mtDAMP release below the threshold that engages innate immune sensors. This statement applies to training rather than to a single bout, and to middle-aged and older adults with or at risk of cardiovascular disease rather than to athletes or young healthy volunteers. The upstream half of the sequence, in which training raises mitochondrial content and antioxidant capacity, rests on human muscle biopsy data. The downstream half, in which reduced mtDAMP release attenuates cardiac and vascular innate immune activation, rests on rodent and cell models. We therefore present mitochondrial quality control as a target worth testing rather than as an established one, and Section 5.1 sets out the measurements that would test it. The settings in which the proposal could be tested to greatest effect are the common cardiovascular conditions listed in Section 4.4 and the populations in whom pharmacological anti-inflammatory treatment is constrained.

The cardiovascular system illustrates how this plays out in disease. Myocardial sterile inflammation runs largely through mtDNA and cGAS–STING; vascular inflammation through oxidized mtDNA and NLRP3. In cardiovascular aging, both axes are active at once, kept coupled by NAD^+^ depletion and the parallel loss of SIRT1 and SIRT3 activity. Exercise acts before these pathways diverge, at the MQC step that controls how much mtDAMP reaches the cytosol. This upstream position is also why exercise suppresses sterile inflammation without the immune compromise observed when downstream effectors such as IL-1β are blocked pharmacologically. A practical implication is that exercise prescriptions and mitochondrial pharmacological agents are best evaluated against mtDAMP readouts rather than against circulating cytokines alone, since cytokine levels reflect events that have already been amplified, while mtDAMP load reflects what is happening at the source.

## Figures and Tables

**Table 2:** Evidence level for each step of the sequence from exercise to mitochondrial quality control to mtDAMP to innate immune activation. Human evidence means the step was measured in human participants or in isolated human cells, animal evidence means it rests on work in intact animals, and in vitro evidence means it rests on isolated cells or tissue of non-human origin. AMPK, AMP-activated protein kinase; cGAS, cyclic GMP-AMP synthase; CMPK2, cytidine/uridine monophosphate kinase 2; IL-1β, interleukin 1 beta; LDL, low-density lipoprotein; LOX-1, lectin-like oxidized LDL receptor 1; mtDAMP, mitochondrial damage-associated molecular pattern; mtDNA, mitochondrial DNA; NLRP3, NOD-like receptor family pyrin domain containing 3; PGC-1α, peroxisome proliferator-activated receptor gamma coactivator 1-alpha; SIRT1, sirtuin 1; STING, stimulator of interferon genes; ULK1, Unc-51-like autophagy-activating kinase 1.

Step in the Framework	What Has Been Shown	Level of Evidence	Section	Ref.
Exercise activates AMPK in contracting skeletal muscle	AMPKα phosphorylation rises in muscle sampled at exhaustion	Human, skeletal muscle biopsy	Section 3.2	[94]
AMPK phosphorylates PGC-1α, and SIRT1 deacetylates it	Direct modification of the coactivator at defined residues	In vitro and animal	Section 3.2	[101,104]
Training raises mitochondrial content and respiratory chain subunit protein	Respiratory chain subunits accumulate progressively across sessions	Human, skeletal muscle biopsy	Section 3.3	[85,89]
Training raises antioxidant defense	Superoxide dismutase and glutathione peroxidase expression rises after four weeks of training and is blocked by antioxidant supplementation	Human, skeletal muscle biopsy	Section 3.4	[87]
Habitual endurance activity is associated with higher mitophagy and fusion protein content	PARKIN and fusion proteins higher in active than in sedentary men	Human, cross-sectional	Section 3.5	[122]
AMPK-dependent ULK1 Ser555 phosphorylation is required for exercise-induced mitophagy	Blocking AMPK in muscle abolishes both the phosphorylation and the mitophagy response	Animal, mouse	Section 3.5	[119]
Improved mitochondrial quality control lowers mtDAMP release	Enhancing mitophagy lowers cytosolic mtDNA and cGAS-STING activation, and blocking autophagy raises them; the two measurements have not been made together in human participants	Animal, mouse; not yet tested in humans	Section 5.1	[7,136]
Cytosolic mtDNA activates cGAS-STING in cardiomyocytes	Cardiomyocyte-specific knockdown or deletion rescues the phenotype	Animal, mouse	Section 4.1	[132,133]
Oxidized mtDNA is required for NLRP3 inflammasome activation	CMPK2 knockdown prevents oxidized mtDNA generation and inflammasome activation	Animal, mouse macrophages	Section 4.2	[9]
Oxidized LDL binding LOX-1 lowers endothelial nitric oxide	An anti-LOX-1 antibody prevents the superoxide rise	In vitro, bovine aortic endothelial cells	Section 4.2	[140]
Neutralizing IL-1β lowers cardiovascular events	Randomized trial with clinical endpoints	Human, randomized trial	Section 4.2	[142]
Training improves clinical outcomes in cardiovascular disease	Randomized trials and meta-analysis	Human, randomized trials	Section 4.4	[92,162,164]
Training lowers mtDAMP-driven innate immune activation in myocardium or vessel wall	Training lowers endothelial NLRP3 induction and pyroptosis; the same readouts have not been obtained from human myocardium or vessel wall	Animal, mouse; not yet tested in humans	Section 4.2	[147]

## Data Availability

No new data were created or analyzed in this study. Data sharing is not applicable to this article.
